# The law of genetic privacy: applications, implications, and limitations

**DOI:** 10.1093/jlb/lsz007

**Published:** 2019-05-14

**Authors:** Ellen Wright Clayton, Barbara J Evans, James W Hazel, Mark A Rothstein

**Affiliations:** 1 Craig-Weaver Professor of Pediatrics, Center for Biomedical Ethics and Society, Vanderbilt University Medical Center, Nashville, TN 37203, USA; 2 Mary Ann and Lawrence E. Faust Professor of Law; Professor of Electrical and Computer Engineering; Director, Center for Biotechnology & Law, University of Houston, Houston, TX 77004, USA; 3 Postdoctoral Fellow, Center for Genetic Privacy and Identity in Community Settings, Vanderbilt University Medical Center, Nashville, TN 37203, USA; 4 Herbert F. Boehl Chair of Law and Medicine, Director, Institute for Bioethics, Health Policy & Law, University of Louisville School of Medicine, Louisville, KY 40202, USA

**Keywords:** DNA, genetics, genomics, GINA, HIPAA, privacy

## Abstract

Recent advances in technology have significantly improved the accuracy of genetic testing and analysis, and substantially reduced its cost, resulting in a dramatic increase in the amount of genetic information generated, analysed, shared, and stored by diverse individuals and entities. Given the diversity of actors and their interests, coupled with the wide variety of ways genetic data are held, it has been difficult to develop broadly applicable legal principles for genetic privacy. This article examines the current landscape of genetic privacy to identify the roles that the law does or should play, with a focus on federal statutes and regulations, including the Health Insurance Portability and Accountability Act (HIPAA) and the Genetic Information Nondiscrimination Act (GINA). After considering the many contexts in which issues of genetic privacy arise, the article concludes that few, if any, applicable legal doctrines or enactments provide adequate protection or meaningful control to individuals over disclosures that may affect them. The article describes why it may be time to shift attention from attempting to control access to genetic information to considering the more challenging question of how these data can be used and under what conditions, explicitly addressing trade-offs between individual and social goods in numerous applications.

## I. INTRODUCTION

People often view genetic information about themselves as private. Each person's genome, or full complement of DNA, is unique,^1^ but the specific variants within an individual's genome may be widely shared with biological relatives or even across the entire human population. This mixed character of the genome—as a uniquely individual assemblage of widely shared common elements—imbues it with a dual private and public significance that confounds any discussion of policy addressing genetic privacy.

On one hand, DNA has been conceptualized as a unique identifier^2^ and a person's book of life,^3^ which provides insights into many aspects of the person's future, although perhaps not as much as many people might think. This conceptualization leads many people to want to control who has access to genetic information about them and drives calls for strong privacy protection or even personal genetic data ownership. On the other hand, genetic data are not limited to one individual, with information about one person revealing information about the person's close and distant biological relatives. Only by studying genetic information from many people can the significance of the individual's variants be discerned. The importance of understanding the causes of health and disease has led some to argue that people have some obligation to share data about themselves for low-risk research.^4^ This public nature and value of the genome makes it difficult to decide what level of control individuals should have and how to provide appropriate privacy protections.

At the same time, the very concept of ‘privacy’ has evolved in recent decades and a new model of privacy has gained ground. The traditional view of privacy as secrecy or concealment—as a ‘right to be let alone’^5^—has grown increasingly strained in the Information Age. The Internet and ubiquitous communication technologies facilitate broad sharing of information, including highly personal information, often without the individual's knowledge or consent.^6^ A new theorization of privacy has emerged, in which concealing one's secrets ‘is less relevant than being in control of the distribution and use by others’^7^ of the data people generate in the course of seeking healthcare, conducting consumer transactions, and going about their lives. ‘The leading paradigm on the Internet and in the “real,’” or off-line world, conceives of privacy as a personal right to control the use of one's data’,^8^ including enjoying access and using it by oneself.^9^

Deciding how much control people should have over access to and use of genetic data about themselves has taken on increased urgency in recent years. Until recently, there simply was less genetic information to worry about, because a person's genetic makeup could be inferred only by studying his or her phenotypic characteristics and family history. It was possible, for example, to tell something about people's eye color genes by looking at their eyes, but not whether they had a gene variant that modestly elevated their cholesterol level or whether they were at increased risk of developing a common complex disorder.

Dramatic advances in technology has now made it possible to examine DNA directly with increasing accuracy and decreasing cost, thereby contributing to the dramatic growth in genome-based approaches, such as exome- or genome-based sequencing, which can provide dramatically more information than single-gene tests. These genomic tests have already proven valuable in diagnosing disorders whose etiology is unknown, as can be the case for some children with developmental disability or critical illness as neonates.^10^ There is also growing interest in using genome-scale tests to answer narrower clinical questions on the ground that these approaches are more efficient than testing a more limited number of genes.^11^ But moving to genome-based technologies has consequences for an individual's privacy because having genomic data makes it possible to examine all the genetic variants regardless of the original reason for testing.

As this technology and our understanding of genomics have improved, a growing number of individuals and entities seek access to individual genetic information. For example, millions of people have pursued testing to learn about their ancestry and to identify previously unknown relatives, endeavors that require access to the information of others as well as their own. In addition, clinicians might seek the data to refine a patient's diagnosis or care. Biomedical researchers might want to examine genetic information to understand the ways that genetic variation contributes to health and disease. Life insurers might want to use this information for underwriting. Parties in toxic tort cases might try to use this information to establish or rebut causation. Law enforcement might want to use the information to identify victims of mass attacks or criminal suspects.

Numerous studies show that many people are more comfortable sharing their genetic data with physicians and researchers in the institution where they seek care than with the government or commercial entities.^12^ People also vary widely in how much they are concerned about genetic privacy^13^ and privacy in general.^14^

Given the diversity of actors and their interests, the increasing power of genetic technologies, and the wide variety of ways these data are held, it is difficult to develop broadly applicable legal principles for genetic privacy. As has been true since the earliest debates about genetic privacy, which began decades ago,^15^ public policy often involves balancing the rights of individuals to maintain the privacy of their genetic information with the rights of other individuals and the public to access the information. The trade-offs often implicate both personal and societal interests, which vary depending on the context. Whether the state can conduct newborn screening for genetic disorders raises different questions from whether an insurer can use genetic information for underwriting health, life, disability, or long-term care insurance, each of which presents its own challenges. In addition, the wide variety of actors and locations are subject to different regulatory schemes.

This article examines the landscape of genetic privacy to identify the roles the law does or should play. Because of the complexity of genetic privacy law, it is infeasible to address all of the issues in a single article. Consequently, the article does not address in detail genetic privacy in reproductive genetic testing,^16^ human subjects research involving genetics, state statutes and regulations pertaining to genetic privacy, and common law actions for invasion of privacy. The article's primary focus is on federal statutes and regulations. After considering the many contexts in which issues of genetic privacy arise, the article concludes that few, if any, applicable legal doctrines or enactments provide adequate protection. For simplicity, and to acknowledge the deep roots of these debates, the article refers to ‘genetic’ privacy, but it clearly contemplates and gives special attention to the implications of the expanding role of genomics and associated technologies.

## II. CONCEPTIONS OF GENETIC PRIVACY

### II.A. Dimensions of Genetic Privacy

In order to understand genetic privacy, it is necessary first to delve into the complex concept of privacy.^17^ Privacy is a state of limited access to an individual or information about an individual.^18^ The right to privacy refers to the ethical and legal principles that recognize the importance of limited access to an individual or information about an individual.

Anita Allen has proposed four categories of privacy applicable to what she terms ‘the ambiguous concept’ of genetic privacy.

When used to label issues that arise in contemporary bioethics and public policy, ‘privacy’ generally refers to one of four categories of concern. They are: (1) informational privacy concerns about access to personal information; (2) physical privacy concerns about access to persons and personal spaces; (3) decisional privacy concerns about governmental and other third-party interference with personal choices; and (4) proprietary privacy concerns about the appropriation and ownership of interests in human personality.^19^

Informational privacy is a particularly important dimension of genetic privacy, and it is the primary focus of this article. From the huge dataset that is every human's genome to family pedigrees and genetic test results, genetics is closely associated with information. Genomics and related analytical approaches—such as proteomics, metabolomics, transcriptomics, and epigenomics—greatly increase the amount of potential gene-associated information about individuals. Often, genetic information is sensitive because it has implications for the current and future health of individuals and their family members. The information may also have major social and economic consequences.^20^

Three other significant concepts within the realm of privacy and genetic privacy are confidentiality, security, and anonymity.^21^ Confidentiality describes a situation in which information is disclosed within a trusting relationship (eg physician–patient) on the express or implied agreement that it will not be divulged to a third party without the permission of the source of the information.^22^ Confidentiality, applicable to the nondisclosure of genetic information,^23^ is a foundational principle in the ethical codes of many health professions and a key element of a wide range of laws. The duty to protect confidentiality is not absolute; however, and in certain circumstances recognized by law or ethical codes, other interests may be paramount, such as the safety and health of third parties.^24^

Security, in the informational sense, is an increasingly important concept in the digital age. It refers to a condition in which individuals or entities with appropriate authority to access certain information are granted access to it, but those without such authority are denied access. Security can be protected by various means, such as by training employees, adopting administrative procedures for handling sensitive information, and implementing technical access controls, including passwords and encryption.^25^

Anonymity is a form of privacy protection in which the identity of the source of certain health information is not obtained or is removed by researchers or other custodians of the information. Anonymization, deidentification, and similar measures are frequently applied to genetic information in an effort to protect individual privacy while retaining the scientific value of the information. The use of anonymized genetic information raises two main concerns. First, technical methods may not be completely effective in preventing the reidentification of genetic information.^26^ Second, there is a plausible argument that individuals’ interest in autonomy should afford them the opportunity to learn about and to control the use of even their anonymized health information or biospecimens.^27^

No matter how people choose to define ‘privacy’, there is a widespread sentiment among legal and ethics scholars that existing privacy laws do not provide as much privacy as many people expect or erroneously believe they have.^28^ US federal privacy laws dating back to the early 1970s strike a balance that grants people some control over their data (through informed consent rights) while also allowing at least some unconsented collection and use of people's data (including their genetic information) for various purposes that lawmakers consider socially beneficial.^29^ The ‘individual control’ these laws provide is thus incomplete. In the 1970s, Congress commissioned a Privacy Protection Study Commission (PPSC) to recommend appropriate privacy protections for many types of data. The PPSC’s 1977 report^30^ acknowledged that unconsented uses of people's data, under certain circumstances, can be ethically justified, but it cautioned that if data cannot be ‘totally protected’ against unconsented access by others, people face privacy risks and need to be able to access their data themselves in order to assess and manage those risks.^31^ Accordingly, many privacy laws, both in the USA and elsewhere, offer individual access rights as a core part of their scheme of privacy protections.^32^ As a practical matter, however, healthcare institutions do not always provide patients with access to their medical records in a timely manner,^33^ and patients often encounter difficulty amending errors in their records.^34^

### II.B. Genetic Exceptionalism

One of the earliest controversies surrounding genetic privacy in the academic literature and policy domain was whether genetic information should be regarded as merely another type of health information or whether certain distinctive characteristics of genetic information demand separate and more protective treatment. Among the allegedly unique aspects of genetic information is the tremendous amount of information contained in DNA, its immutability, its potential use as a unique identifier, and its implications for family members and others with a similar geographic ancestry.

Thomas Murray, recalling a debate in the 1980s about whether HIV information was unique (termed ‘HIV exceptionalism’), coined the term ‘genetic exceptionalism’ in reference to the controversy surrounding whether genetic information—at that time typically referring primarily to Mendelian or single-gene disorders—should be treated separately.^35^ Murray also recognized that the main difference between genetic and nongenetic information is that many members of the public regard anything ‘genetic’ as special. ‘Genetic information is special because we are inclined to treat it as mysterious, as having exceptional potency or significance, not because it differs in some fundamental way from all other sorts of information about us’.^36^ A practical problem with the separate treatment of genetic information is the difficulty in defining and separating it from other medical information in health records.^37^ Separate treatment of genetic information also contributes to genetic reductionism^38^ and genetic determinism,^39^ thereby increasing rather than reducing the seeming importance of genetic information and the stigma of genetic disorders.

As with other types of information in emerging medical fields, many of the problems associated with the use of genetic information arise from two time lags. First is the time lag between the discovery of a genetic basis for a condition and the development of therapies to prevent, treat, or cure the disorder. Thus, genetic information may indicate a risk, such as for Alzheimer's disease, about which little or nothing can be done to prevent or ameliorate the condition. Second is the time lag between a genetic test that identifies the increased risk of disease in a particular individual and the onset of symptoms. During this time period, when the individual is in medical limbo, numerous entities with an economic interest in the individual's future health, such as various insurance companies, are inclined to use the genetic information to limit their risk. Neither of these characteristics is unique to genetics.

Although most commentators have been critical of genetic exceptionalism,^40^ virtually all of the recent legislation enacted to deal with genetic privacy and genetic discrimination has been genetic-specific. One of the main reasons for this choice is that genetic-specific laws are necessarily narrower in scope and are thus more likely to garner political support. For example, as early as the 1970s, a few states began enacting laws prohibiting some types of genetic discrimination in health insurance.^41^ These laws provided additional protections to those afforded by state medical privacy laws, which also have numerous exceptions.^42^ Congress enacted the Genetic Information Nondiscrimination Act (GINA) in 2008,^43^ but its prohibition against genetic discrimination in health insurance applies only to asymptomatic individuals. It was not until 2010 that Congress prohibited all health-based discrimination in health insurance when it enacted the Affordable Care Act.^44^ This universally applicable nondiscrimination law provides comprehensive protections and avoids coverage gaps that characterize genetic nondiscrimination laws.

From a policy perspective, advocates and elected officials often have to decide whether to accept limited, genetic-specific legislation or to hold out for the possibility of a broader statute. On balance, less protective genetic laws are better than no legislation at all only if the enactments provide some clear improvement over the status quo, are drafted carefully to avoid unintended consequences, including reifying genetic exceptionalism, do not delay enactment of more comprehensive legislation, and are not presented to the public as a complete answer to the problem.^45^ Thus, advocates and policy-makers often are forced into an unappealing choice between limited, genetic-specific legislation or no legislation at all. Whether it is better to enact weak genetic privacy protections, as opposed to holding out for broader and more forceful privacy legislation, depends on several factors. For example, will passage of weak and incomplete genetic privacy protections reduce pressure for the stronger protection or lull the public into a false belief that their genetic information is better protected than it actually is?

## III. GENETIC INFORMATION IN HEALTHCARE

Genetic information connected to personal identifiers is generated and used in a variety of contexts that may or may not be health-related—eg, clinical genetics, direct-to-consumer (DTC) testing,^46^ and forensics.^47^ Genetic information is an essential clinical tool in an increasing number of medical specialties, including clinical genetics, oncology, obstetrics, neurology, pediatrics, and behavioral health. As clinicians obtain, aggregate, store, use, and disclose more genetic information, there is a greater possibility of breaches of privacy, confidentiality, and security. Some scenarios where such breaches may occur include the following: (1) genetic information is disclosed to or accessed by healthcare providers without the authority or legitimate need to see it; (2) the scope of the genetic information obtained and disclosed is beyond that needed for a legitimate healthcare purpose; and (3) genetic information is used for a purpose unrelated to the disclosure.^48^ Each of these, and many other situations in clinical settings, raises important legal and ethical issues.^49^

Uses and disclosures of health (including genetic) information in healthcare settings raise several issues, including whether consent or authorization is required, how much and what type of information can lawfully be disclosed, and which members of the treatment or research team should have access to which information. Whereas individuals are often concerned about discrimination when their health information is disclosed beyond healthcare settings, in healthcare settings their main concerns are protecting their privacy, autonomy, and dignity. Even though these concerns may seem abstract or indirect, many individuals regard them as very important, and concerns about these issues often influence a patient's behavior and health outcomes, such as where patients limit disclosures of sensitive information to their healthcare providers to protect their privacy.^50^

### III.A. HIPAA Privacy Rule

Most disclosures in healthcare settings are by ‘covered entities’ under the Health Insurance Portability and Accountability Act (HIPAA)^51^ and its Privacy Rule.^52^ HIPAA was enacted in 1996, primarily as an insurance statute, to facilitate the movement of employees from one employer to another without interruption or loss of employer-sponsored group health coverage for the employee or the employee's dependents. Its role as privacy legislation was something of an afterthought. Congress added ‘Administrative Simplification’ provisions^53^ to HIPAA during the legislative process to mandate the use of standard electronic formats in the submission of health insurance claims; these provisions addressed privacy only insofar as needed to minimize privacy risks related to the electronic filing of insurance claims. Thus, the HIPAA statute gave the US Department of Health and Human Services (HHS) the jurisdiction to regulate entities that provide healthcare or pay for it (such as insurers) but gave HHS no jurisdiction to regulate the multitude of other private companies and institutions (eg drug manufacturers, research institutions that provide no healthcare services, companies that sell fitness-tracking devices, DTC genetic testing services, and many others) that—in our current times—use and store people's health and genetic data in ways that affect their privacy.

Congress understood that the HIPAA statute did not grant HHS the jurisdiction it really needed to be an effective health or genetic privacy regulator. Accordingly, HIPAA envisioned that Congress would subsequently enact broad national health privacy legislation by August 21, 1999.^54^ HIPAA gave HHS the authority to promulgate the HIPAA Privacy Rule only if Congress failed to legislate by that date.^55^ As events unfolded, Congress did not enact the new privacy legislation and it fell on HHS to do the best it could with the limited jurisdiction available under the HIPAA statute. Consequently, the Privacy Rule applies only to four types of HIPAA-covered entities involved in the payment chain of healthcare: (1) healthcare providers that transmit any health information in electronic form in connection with a covered transaction; (2) health plans, including a health insurer, HMO, Medicare or Medicaid program, or other entity that provides or pays the costs of medical care; (3) health clearinghouses, public or private entities, including a billing service or health information management system, that process health information into a standard format for billing purposes; and (4) business associates of these entities, including individuals or entities that perform or assist in billing, management, administration, or other functions regulated by the Privacy Rule.^56^ The Privacy Rule was never intended to be a comprehensive health privacy regulation, but it has assumed such a role by default because of Congress's failure to enact more sweeping and rigorous health and genetic privacy laws and regulations.^57^

Other than a definitional provision^58^ that Congress ordered HHS to add to the Privacy Rule under GINA,^59^ a provision dealing with deidentification,^60^ and two provisions dealing with health plans,^61^ the Privacy Rule does not contain any special provisions for genetic information.^62^ Under GINA, genetic information is deemed to be ‘health information’ that is protected by the Privacy Rule^63^ even if the genetic information is not clinically significant and would not be viewed as health information for other legal purposes. In other words, the Privacy Rule rejects genetic exceptionalism and places genetic information under the ordinary protections of the HIPAA Privacy Rule.^64^ The Privacy Rule provides that a covered entity need not obtain consent or authorization from the individual for uses and disclosures of protected health information (PHI)^65^ (individually identifiable health information) for treatment, payment, or healthcare operations.^66^ A covered entity is merely required to include information about its uses and disclosures in a notice of privacy practices provided to all individuals.^67^ The Privacy Rule also has glaring gaps in its framework for keeping people informed about who has been given access to their genetic information. For example, when a person's genetic information is disclosed in a deidentified format, the Privacy Rule's ‘accounting of disclosures’ provisions^68^ do not require covered entities to tell the individual about the disclosure, even though deidentified genetic information is potentially reidentifiable.

An important privacy-enhancing element of the Privacy Rule is the minimum necessary provision, which states that uses and disclosures of PHI for payment and healthcare operations must be limited to ‘the amount reasonably necessary to achieve the purpose of the disclosure’.^69^ This provision, however, is not applicable to disclosures for treatment.^70^ Furthermore, for treatment, payment, and healthcare operations, there is no requirement that covered entities use and disclose PHI in the least identifiable form consistent with legal requirements or the purpose of the use or disclosure.^71^

Besides the HIPAA Privacy Rule, several states have enacted ‘genetic privacy’ laws, which vary widely in their applicability and stringency. For example, some of these laws require informed consent for genetic testing, regulate access to genetic information, or provide that genetic information is the property of the individual.^72^

### III.B. GINA

In 2008, after 13 years of contentious congressional deliberation, GINA was overwhelmingly passed by Congress and signed into law by President George W. Bush.^73^ Unlike other civil rights laws, GINA was not enacted to remedy ongoing discrimination; rather, it was intended to preempt discrimination that was feared, but not well documented as yet occurring.^74^ Section 2(5) of GINA confirms that the purpose of the law is ‘to fully protect the public from discrimination and to allay their concerns about the potential for discrimination, thereby allowing individuals to take advantage of genetic testing, technologies, research, and new therapies’. GINA’s two main titles prohibit discrimination based on genetic information in health insurance (Title I) and employment (Title II), but the value of this legislation has been a source of some dispute.^75^

Although GINA is best known for its provisions prohibiting discrimination based on genetic information, it also contains provisions related to privacy. Section 202(b) of GINA prohibits employers from requesting, requiring, or purchasing genetic information with respect to an employee (including an applicant) or a family member of the employee. Similar provisions limiting the acquisition of genetic information are included in Title I dealing with nondiscrimination in health insurance and health benefit plans.^76^

Section 105 of GINA also provides that genetic information—as broadly defined by GINA^77^—‘shall be treated as health information’ under HIPAA, thereby extending the HIPAA Privacy Rule to genetic information regardless of whether it is ‘health information in the ordinary sense of this word’.^78^ This seeming expansion of the Privacy Rule is subject to important limitations. First, as noted above, the Privacy Rule only applies to covered entities in the healthcare payment chain, and it does not apply to many other entities that acquire, store, use, or disclose genetic information, such as insurers other than health insurers. It also does not generally apply to DTC genetic testing companies, including ancestry testing companies. The second limitation is that the Privacy Rule notoriously contains numerous exceptions to its individual authorization requirements, discussed below. Third, many observers view its protections as inadequate because it is enforceable only by HHS’s Office for Civil Rights and does not create a private right of action on behalf of the person whose data are disclosed.^79^ Therefore, the nominal privacy protection afforded to genetic information in the possession of HIPAA-covered entities does not fully address the need for genetic privacy protections.

### III.C. ACMG List

One of the most controversial issues surrounding disclosure of genetic information in healthcare settings involves what genetic information healthcare providers (eg clinical geneticists, genetic counselors) can and should look for and share with their patients beyond that needed to address the patients’ immediate clinical question. A key issue is whether there is a professional obligation to provide secondary findings of genome sequencing for a predetermined set of gene variants. The American College of Medical Genetics and Genomics (ACMG) originally adopted the position that, because of the significance of certain results, it is mandatory that professionals performing the sequencing, interpretation, or disclosure of the results in clinical settings include 57 medically actionable genes, regardless of the wishes of the patient or ordering physician, or their pertinence to the patient's clinical problem.^80^ This position was widely criticized as violating patient autonomy and clinician discretion.^81^ The ACMG subsequently amended its policy to provide that patients could decline to receive secondary results.^82^

### III.D. Informing At-Risk Relatives

A related issue involves the ethical and legal obligations of clinicians to offer information about a patient's diagnosis of a gene-mediated disorder or the results of a genetic test to at-risk family members. There is widespread agreement that clinicians should advise their patients about the importance for their relatives of significant diagnostic or predictive genetic information. Ideally, the clinician would encourage disclosure and offer to assist the patient in this process, but there has been disagreement about whether clinicians have a duty to contact and offer the results to relatives when the patient refuses and does not authorize the clinician to contact them. A much-discussed judicial opinion suggested that there might be a legal duty for a physician to make these disclosures to a patient's relatives,^83^ and a guidance document from the American Society of Human Genetics stated that disclosure is appropriate in certain highly unusual circumstances.^84^ Nevertheless, both of these sources predated the 2003 compliance date of the HIPAA Privacy Rule, which prohibits nonconsensual disclosure of genetic information to relatives of a patient.^85^ Furthermore, imposing such a duty might discourage individuals from obtaining genetic testing, cause an irreparable rift between patients and their healthcare provider, prove to be burdensome and infeasible in identifying and contacting the patient's relatives, and result in harm by offering to disclose sensitive health information that the relatives might not want to receive. Therefore, as a matter of ethics and law, clinicians are neither required nor permitted to inform the genetically at-risk relatives of their patients without the consent or authorization of their patient or their patient's personal representative.^86^ The disclosure of research results raises similar issues.^87^

## IV. GENETIC INFORMATION IN DTC GENETIC TESTING

Outside the healthcare setting, millions of people now obtain DTC genetic testing for a wide range of purposes, some of which can impinge on their privacy interests or the privacy interests of others. Companies now purport to provide genetic insights into health, ancestry and genealogy, family relationships, and lifestyle choices.^88^ They offer advice about using genetic test results to guide choices about food and dieting, selection of sports purportedly based on physiologic traits correlated with athletic ability, or even how to pick a partner or where to travel. The majority of these companies do their own genetic testing, but a few ask customers to upload test results they have obtained elsewhere for further analysis.

The most prevalent categories of DTC genetic tests consist of those designed to provide insights into ancestry and family relationships.^89^ Although some people seek primarily to learn about their ancestral origins, others hope to find blood relatives whom they had not previously known about. Still others have desires that may be more disruptive, such as to identify the birth parents of a child who was adopted, or a gamete donor,^90^ which may lead to unwanted contact,^91^ or to identify the parentage of a child, which may be done surreptitiously and the results of which can have significant legal consequences for children and adults. All of these efforts to define biological relationships require people to share their genetic data.

Companies are also beginning to provide genetic tests that can be broadly understood as health-related, directly to the consumer and without the involvement of a healthcare provider. Recent regulatory developments have been driven largely by the Food and Drug Administration (FDA) and 23andMe, which became the first company authorized to market a DTC carrier test for Bloom Syndrome in 2015.^92^ 23andMe subsequently obtained authorization to market Genetic Health Risk (GHR) tests for 10 conditions in 2017, including Parkinson's disease and late-onset Alzheimer's disease,^93^ followed by a GHR report for selected variants of BRCA1/BRCA2 in 2018.^94^ Under this new regulatory approach, the FDA ‘intends to exempt additional 23andMe GHR tests from the FDA’s premarket review, and GHR tests from other makers may be exempt after submitting their first premarket notification […] allow[ing] other, similar tests to enter the market as quickly as possible and in the least burdensome way, after a one-time FDA review’.^95^ Most recently, in October of 2018, the FDA authorized 23andMe to market a Pharmacogenetic (PGx) Reports test that detects 33 genetic variants associated with medication metabolism (eg response to certain antidepressants and cardiac medications), imposing a warning label requirement designed to inform consumers that they should not make any changes to their medications based on the results.^96^

A 2017 study of 90 DTC-GT companies operating within the USA sheds light on the information that these companies provide to consumers about their genetic data practices.^97^ Although industry leaders generally had fairly comprehensive policies, almost 40% of the companies surveyed (35 of 90) provided no information to consumers about their genetic data practices, including the fate of biological samples or the resulting genetic data. Of the 55 companies with policies governing genetic data, just over half stated what information would be shared with the testing laboratory or what procedures, if any, were used to safeguard the information. Only half discussed whether the sample would be stored or not, a number of which had a policy of retaining the physical sample (eg a saliva sample, cheek swab, or the extracted DNA). In addition, many indicated that they would retain any genetic data generated from these samples indefinitely. While most policies made vague guarantees or assurances about data security, very few provided specific details, and almost none stated that they would notify customers in the event of a breach.

Policies also varied in terms of what information was provided regarding ownership and commercialization of genetic data. Many companies did not explicitly claim ownership of a consumer's DNA, but they often retained broad rights to commercialize the resulting data. Of the 55 companies with policies governing genetic data, nearly half (23 companies) had policies with provisions that indicated data would (or might) be shared with third parties, yet none provided an exhaustive list. Eighteen explicitly stated that they would share deidentified data with third parties without further consent. Ten companies allowed participants to opt-in for sharing data with outside researchers, while five explicitly permitted such sharing by default. The majority of the 38 companies that addressed sharing data with the government or law enforcement said only that they would do so ‘as required by law’ (eg in response to a subpoena, court order, regulation, or statute), but they provided little or no information about how they would handle such a request. In addition, many policies contained broad ‘catch-all’ provisions that provided for disclosure to third parties beyond law enforcement under a variety of circumstances.^98^

The shortcomings of these policies in defining what data will be retained and with whom they might be shared are particularly worrisome because these companies typically are not subject to many of the laws that apply in clinical settings, such as HIPAA^99^ and Clinical Laboratory Improvement Amendments (CLIA).^100^ As discussed above, the FDA has asserted authority to regulate only companies like 23andMe that provide certain health-related tests. The rest of the industry is largely left to self-regulate, including with respect to the quantity and quality of information they provide to consumers about their company's genetic data practices.

State laws may also implicate the DTC industry, but they vary widely by jurisdiction and in their scope. States regulate through a variety of mechanisms, some of which are specific to genetic testing and the resulting data, including laboratory licensing requirements, defining what constitutes the practice of medicine and who is authorized to order certain genetic tests, or imposing informed consent requirements.^101^ A small subset of states also grant individuals a property interest in their genetic information.^102^ Other laws are directed at the e-commerce industry more broadly but may also implicate DTC services. ^103^ While state law may provide consumers with potential causes of action against DTC companies in certain circumstances,^104^ these efforts are complicated by the fact that consumers typically agree to terms and conditions that contain exclusion clauses that limit a company's liability or provisions that limit the remedies and damages available to the consumer.^105^

A relatively low baseline of protection is provided by the Federal Trade Commission (FTC), which has broad authority to police ‘unfair’ or ‘deceptive’ business practices under the century-old Federal Trade Commission Act.^106^ Despite this authority, the FTC has rarely taken action against DTC genetic testing companies. The only meaningful enforcement action to date occurred in 2014, against GeneLink, Inc., on the grounds that its health-related claims of benefit were not supported by the evidence and that its data security practices deviated from its privacy policy in such a way as to rise to the level of unfair and deceptive.^107^ It is troubling that this is the only enforcement action, because many DTC genetic companies fail to provide adequate information regarding how genetic information will be collected and retained, how it will be used by the company, or with whom it will be shared, practices that would appear to be at odds with the FTC’s articulation of the Fair Information Practice Principles (FIPPs)^108^ and the agency's Proposed Privacy Framework.^109^

In the absence of a robust regulatory framework or binding guidelines governing genetic data practices, the DTC genetic testing industry is left to develop its own voluntary best practices. In 2018, the Future of Privacy Forum released ‘Privacy Best Practices for Consumer Genetic Testing Services’, a document produced in coordination with leading DTC genetic testing companies (23andMe, Ancestry, Helix, MyHeritage, and Habit) and consumer and privacy advocates.^110^ The Best Practices, which incorporate feedback from the FTC and draw heavily on the FIPPs, consist of eight principles designed ‘to address the privacy issues related to the collection, retention, use, sharing, and research based on Genetic Data’: (1) transparency; (2) consent; (3) use and onward transfer; (4) access, integrity, retention, and deletion; (5) accountability; (6) security; (7) privacy by design; and (8) consumer education.^111^ It is worth noting that these guidelines do not place restrictions on genetic data that have been deidentified if ‘the deidentification measures taken establish strong assurance that the data is not identifiable’.^112^

Although adoption of the Best Practices is voluntary, and thus lack an enforcement mechanism, companies are encouraged to ‘[p]rovide public/consumer facing commitments that are enforceable by the FTC, State Attorneys General, or other authorities’.^113^ The industry efforts embodied in the Best Practices represent a positive development and help to facilitate a dialogue about important privacy issues, but it remains to be seen whether they will be widely adopted across the diverse DTC-GT industry. It is also unclear whether companies will be willing to make disclosures not currently mandated under existing laws and regulations, especially disclosures that could expose a company to potential liability.^114^

## V. OTHER USES AND DISCLOSURES OF GENETIC INFORMATION

For individuals to maximize the healthcare benefits of their genetic data generated by research (eg All of Us), DTC genetic tests, and other sources, the information needs to be submitted and entered into the individual's health record. Once in an electronic health record (HER), however, it is subject to various nonconsensual disclosures permitted by the HIPAA Privacy Rule as well as numerous other disclosures compelled by entities with the legal and/or economic leverage over the individual to require the individual to execute a HIPAA-compliant authorization. According to a recent estimate, each year in the USA there are at least 25 million compelled disclosures of health information for various purposes, such as applications for employment and life insurance.^115^ Many of these authorizations are not limited in scope or otherwise do not prohibit redisclosure of the information to other entities.

### V.A. HIPAA Public Purpose Exceptions

The HIPAA Privacy Rule contains 12 ‘public purpose’ exceptions, which permit covered entities to disclose PHI, including genetic information, without the authorization or consent of the individual. These provisions permit the following uses and disclosures: (1) required by law;^116^ (2) for public health activities;^117^ (3) about victims of abuse, neglect, or domestic violence;^118^ (4) for health oversight activities;^119^ (5) for judicial and administrative proceedings;^120^ (6) for law enforcement;^121^ (7) about decedents;^122^ (8) for cadaveric organ, eye, or tissue donation;^123^ (9) for some types of research;^124^ (10) to avert a serious threat to health or safety;^125^ (11) for specialized government functions, including national security;^126^ and (12) for workers’ compensation.^127^ The Privacy Rule does not require any disclosures under this provision. Any requirement for covered entities to disclose information, such as to notify public health agencies about certain infectious diseases, arise under separate provisions of federal or state law. The public-purpose exceptions to the Privacy Rule establish that disclosure of PHI for such a purpose is ‘permissive’ in the sense that covered entities may make such disclosures without violating the Privacy Rule.

### V.B. Other Lawful Uses of Genetic Information

Beyond the HIPAA public purpose exceptions, there are numerous instances in which genetic information may be of great interest to other individuals or entities beyond the healthcare setting. Arguably, the greatest threat to informational health privacy is the fact that disclosure of health information (often including genetic information) may be required as a lawful condition of a transaction or an application for benefits and that the information is no longer protected under federal law once disclosed to an entity not covered under the Privacy Rule.^128^ Generally, the two main concerns in compelled disclosures are the scope of the information disclosed and whether the use of the information can result in discrimination.^129^ The following common uses of genetic information generally involve instances in which consent or authorization is not legally required or may be compelled by a third party seeking the information.

#### V.B.1. Criminal Justice and Forensics

Various federal and state statutory provisions apply to the use of genetic information in criminal justice. The Combined DNA Index System (CODIS), the federal system for the collection, analysis, storage, and use of DNA samples for forensic purposes, was established by the DNA Identification Act of 1994.^130^ Through a tiered system of databases, ‘CODIS enables federal, state, and local crime laboratories to exchange and compare DNA profiles electronically, thereby linking crimes to each other’ and to individuals whose DNA profiles are in CODIS.^131^ The success of DNA forensic identification programs has led to calls for expanded collection and searching, such as proposals for population-wide databases^132^ and the use of partial matches (or ‘familial searches’).^133^ Besides forensic identification, behavioral genetic information might be used at other stages of the criminal justice system, such as at a bail hearing as evidence of flight risk, at a trial on the issue of criminal capacity, and at parole hearings on the issue of the likelihood of recidivism.^134^ The introduction of unvalidated behavioral genetic theories, however, risks encouraging behavioral genetic reductionism and determinism. Also of importance to genetic privacy, the HIPAA Privacy Rule provision permitting covered entities to disclose PHI for law enforcement does not require a warrant, subpoena, or any other legal process prior to disclosure.^135^

#### V.B.2. Education

Federal privacy protection extends to health information, including genetic information, collected, stored, or used by educational institutions under the Federal Educational Rights and Privacy Act.^136^ Other laws applicable to the use of genetic information in education include the Individuals with Disabilities Education Act,^137^ Title II of the Americans with Disabilities Act (ADA),^138^ and section 504 of the Rehabilitation Act.^139^ Although little predictive genetic information is currently used in educational settings, in the future student genetic information might be used (or misused) in admissions, educational placement, curriculum development, and discipline.^140^

#### V.B.3. Employment

Title II of GINA prohibits discrimination in employment on the basis of genetic information.^141^ The law, applicable to employers with 15 or more employees, attempts to prevent discrimination by restricting access to or use of genetic information about applicants, employees, and their family members.^142^ GINA must be read in conjunction with Title I of the Americans with Disabilities Act (ADA), which prohibits discrimination in employment on the basis of disability. Section 102(d)(3) of the ADA provides that after a conditional offer of employment an employer may require a conditional offeree to submit to an ‘employment entrance examination’, which may be of unlimited scope, and also to execute a HIPAA-compliant authorization for the release of all of the individual's health records.^143^ After GINA, this provision on medical examinations and disclosures applies to all health information except genetic information.^144^ The problem is that it is difficult to segregate genetic information in medical records, especially because the definition of genetic information in GINA includes family health histories. Therefore, it is common for healthcare providers to disclose complete health records, which often includes genetic information.

Another problem with GINA is that it applies only to individuals whose genetic condition has not ‘manifested’ and therefore are asymptomatic. On the other hand, the ADA provides a remedy for individuals who have been subject to discrimination based on expressed genetic conditions that cause a substantial limitation of a major life activity. Again, reading GINA and the ADA together, individuals who have a manifested genetic condition that does not constitute a substantial limitation of a major life activity are not protected by either law.^145^ In addition to federal laws, 35 states have enacted laws prohibiting genetic discrimination in employment.^146^

#### V.B.4. Family Law

State laws traditionally regulate virtually all aspects of family law, including adoption, child custody, and paternity determinations. DNA forensic tests have revolutionized the proof of paternity,^147^ although some uses of the technology are not necessarily beneficial to children, such as disestablishment lawsuits brought by nonmarital fathers seeking to end their support obligations.^148^ The Uniform Parentage Act, most recently revised in 2017 by the Uniform Law Commission, attempts to regularize the rules for acknowledgments, denials, notifications to presumed fathers, and other issues.^149^

#### V.B.5. Government Benefits

The availability of several types of government benefits depends on the proof of the cause of a claimant's injury or disability. Genetic information, along with other medical information, may be used to establish the etiology of a health condition. For example, genetic information may help to prove or disprove the service-relatedness of a claim for veterans’ benefits or the work-relatedness of a workers’ compensation claim.^150^

#### V.B.6. Immigration

Family reunification has been an important principle of international immigration law since the Universal Declaration of Human Rights in 1948.^151^ Several developed countries have used DNA testing to establish genetic relatedness,^152^ although some immigrant organizations claim that such testing is expensive and has been used to discourage immigration from ‘undesirable’ countries.^153^ Requiring genetic connections also disadvantages those whose family relationships are based on adoption or alternative reproductive technologies as well as more informal kinship/care giving relationships. It is unclear whether DNA testing will have an increasingly important role in the USA as a way to verify the relatedness of immigrants and asylum seekers.

#### V.B.7. Insurance

Genetic discrimination in insurance, especially health insurance, was one of the first public concerns raised by the Human Genome Project.^154^ By the end of the 1990s, 48 states had enacted laws prohibiting genetic discrimination in health insurance.^155^ GINA, enacted in 2008, added federal protection, but like the state laws, it only prohibits discrimination against asymptomatic individuals. The Affordable Care Act,^156^ by prohibiting all health-based discrimination in individual and group health insurance, provides more comprehensive nondiscrimination protection. Some states also regulate the use of genetic information in other insurance products,^157^ including life,^158^ disability,^159^ and long-term care insurance,^160^ but none of them prohibits the underwriting use of an individual's genetic information contained in his or her health records.^161^

Regulating the use of genetic information in insurance is extremely difficult for several reasons. First, the social function of insurance varies greatly among the various types of insurance products. Second, the insurance industry is large, politically powerful, and, in the case of life insurance, has been doing business largely the same way for centuries. It is loath to make fundamental changes in underwriting practices, including unlimited access to applicants’ health information, which the industry believes is necessary to prevent adverse selection. Third, there is a close relationship between private insurance and public programs for income replacement. For example, regulatory changes in disability insurance underwriting would affect government expenditures for Social Security Disability Insurance and regulatory changes in long-term care insurance underwriting would affect government expenditures for Medicaid payments for nursing home care. If insurers could deny coverage to genetically at-risk individuals, the increased costs for these individuals would be borne by taxpayers rather than other insurance policyholders.

#### V.B.8. Occupational and Environmental Health

Individuals vary widely in their susceptibility and response to occupational and environmental toxins, and toxicogenomics has helped to explain the genetic basis of many of these differences.^162^ The use of genetic and genomic information in occupational and environmental risk assessment raises numerous issues, including setting the most appropriate exposure limits, establishing the duties owed to sensitive individuals, defining the relationship between regulatory and nondiscrimination statutes, and balancing the roles of autonomy and paternalism in deciding whether individuals should be able to accept increased risks.^163^ GINA prohibits the use of genetic information in employment decisions,^164^ but genetic information is likely to play an increased role in regulating exposures covered by the Occupational Safety and Health Administration and the Environmental Protection Agency.

#### V.B.9. Personal Injury Litigation

Genetic information can play an important part in personal injury litigation. Besides medical malpractice cases, genetic information might be relied upon by either plaintiffs or defendants in attempting to prove or disprove causation in toxic tort and other cases involving allegedly harmful exposures.^165^ In any personal injury case in which a court is asked to base prospective damages on the life expectancy of the plaintiff, the defendant may seek to compel genetic testing of the plaintiff or to admit predictive genetic information into evidence.^166^ In such an event, already-injured plaintiffs may be forced to learn genetic information that they would prefer not to know.

#### V.B.10. Real Property and Commercial Transactions

Genetic discrimination claims involving real property and commercial transactions are likely to grow in importance. For example, senior residential communities, mortgage companies, or other entities might seek to prevent individuals with a genetic predisposition to Alzheimer's disease from purchasing, renting, or obtaining financing for real property.^167^ A retirement facility might be concerned that individuals with Alzheimer's disease would undermine the development's marketing strategy of appealing to vibrant, active, and healthy retirees. It is not clear whether the federal Fair Housing Act,^168^ as amended, which prohibits discrimination based on disability, would apply to genetic discrimination. California is the only state that specifically prohibits the use of genetic information to discriminate in housing.^169^

## VI. GENETICS AND IDENTIFICATION

Genetic data are in identifiable form in a patient's EHR. The privacy issues, then, are who can get access to these records as well as what can be done with the data once they have been obtained. Access to information in the medical record is controlled primarily by HIPAA, which as noted above has numerous exceptions, as well as state law in some jurisdictions. Data collected or used in National Institutes of Health (NIH)-funded research has additional protections. To comply with the 21^st^ Century Cures Act,^170^ the NIH now automatically issues Certificates of Confidentiality, which prevents compelled disclosure to most third parties, to all NIH-funded research involving ‘identifiable, sensitive information’, specifically defined by NIH as including ‘[r]esearch that involves the generation of individual level, human genomic data from biospecimens, or the use of such data’.^171^ Use of genetic data is also subject to anti-discrimination laws, such as GINA, the ADA, and some state laws, as well as provisions of the ACA.

### VI.A. The Debate about Reidentification

Deidentification and reidentification of genetic specimens is a contentious issue. A valuable starting point to the policy debate is asking how likely is it that people will be harmed by being identified from genetic data from which identifiers have been removed, which commonly occurs in research. Deidentification is often done to protect the identity of research participants and their families.^172^ Researchers also may seek to deidentify data to facilitate their investigations, eg, to avail themselves of the exception to the Common Rule ‘if the information is recorded by the investigator in such a manner that subjects cannot be identified, directly or through identifiers linked to the subjects’^173^ and to avoid the need to obtain authorization under the HIPAA Privacy Rule.^174^ The practice of deidentifying data for research has a long history, particularly in epidemiological studies, of which modern genomics is a part.

Although some have worried for years that genomic data are particularly identifiable because they are unique,^175^ there has been no tsunami of efforts to reidentify people from their DNA or genomic data.^176^ This result was to be expected in the context of research because research institutions have strong incentives to provide security for data in order to avoid federal and state penalties as well as bad publicity. It is common practice to require that investigators contractually agree not to attempt to reidentify the individuals from whom data were derived, and some institutions audit researchers to ensure that this does not occur.^177^

Perhaps more important, identifying the source of an unknown sample of DNA or genetic data typically requires that it be matched to an identified sample, either directly or through familial tracing,^178^ ie, the identification of individuals who share DNA sequences with the targeted individual. Until recently, the main sources of identified genetic data in the USA were forensic databases, which are accessible only to law enforcement. These data rely on a limited number of noncoding, short tandem repeats (STRs), a different DNA characteristic from those historically contained in research datasets, which often focus on analysing single base pair changes.^179^ While STR results are shared among law enforcement in the CODIS system, identifying information is retained locally. Moreover, the limited number of markers in forensic databanks limits the power of familial tracing to close relatives.

What has changed is the convergence of the dramatically decreased cost of sequencing and data storage, the increased ease of sharing data on the Internet, and the rise of new business services that offer analysis and interpretation of DNA sequence data. Millions of people have submitted samples for analysis to DTC companies. These companies advertise a wide array of products, ranging from providing health information to uncovering family relationships for genealogy or detecting misattributed parentage. These companies vary in what analysis they perform, but many examine hundreds of thousands of single nucleotide polymorphisms (SNPs), or single base pair changes, yielding a tremendous amount of data. These data, because of their size, make it possible to identify far more distant relatives than can be achieved using forensic databases.

Thus, the likelihood of being reidentified often turns on the extent to which these commercial or public repositories control access to the data they hold. The largest companies, 23andMe and Ancestry.com, strive to protect the identity of their customers, for instance, by asking customers whether they want to reveal their identity to a putative relative. Moreover, these two companies have vigorously resisted requests for access by law enforcement, efforts they make public in their transparency reports.^180^ A recent article by Hazel and Slobogin, however, reveals that most sites, including the large number that engage in nonconsensual, surreptitious testing, have poor privacy policies at best.^181^ Thus, these companies may be ready sources of identified genomic data.

Companies’ policies are not the only factor increasing the possibility of reidentification, as millions of people have posted genomic data with identifiers on open access websites. Some place these data on such sites as the Personal Genome Project^182^ or OpenSNP.org.^183^ Interestingly, some of the latter's depositors still believe their privacy is protected.^184^ The site hosting individually identified genetic data that have received the most attention recently, however, is GEDMatch,^185^ a citizen-run site created to facilitate genealogy research in which over one million people have placed their identified raw SNP data from DTC companies. Indeed, some investigators have opined that ‘a large percentage of people have at least one high-confidence genetic cousin in GEDmatch’.^186^ Until recently, that site's privacy policy read, in part:

While the results presented on this site are intended solely for genealogical research, we are unable to guarantee that users will not find other uses. If you find the possibility unacceptable, please remove your data from this site.^187^

Data from this site were used by law enforcement to identify the infamous Golden State Killer, by identifying and then tracing a fourth cousin.^188^ Since that identification, another forensics company reportedly has submitted samples from 100 cases to GEDMatch and has identified 20 close matches. The founders of GEDMatch report that more people support the use of data to identify potential criminals than object.^189^ Nonetheless, they have updated the site's privacy policy, noting that data may be used for familial searches to identify perpetrators. Interestingly, they state that law enforcement is specifically permitted to upload ‘raw data’ to identify perpetrators of sexual assault or homicide. They define this as a new limit on what the police can do, implicitly rejecting access to solve other types of crimes. Their new policy requires that people submitting data about third parties have permission or legal authority to do so,^190^ although how this would be enforced is by no means clear.

In the future, it may be possible to infer enough about an individual's facial features from his or her DNA^191^ to permit the person to be identified, especially in light of the growing sophistication of photograph tagging software. How well such predictions work currently, however, has been questioned.^192^ Nonetheless, the Bavarian parliament recently enacted a controversial law permitting law enforcement to analyse DNA to predict phenotypic characteristics such as eye color to assist in their investigations.^193^

Additional risks that people can be identified from research, clinical information, or biospecimens arise because most genomic research involves other data about participants, including their demographics,^194^ medical history, their activities, and their social and built environment. These other data can be more easily identifiable in the current data environment than are genomic data themselves.^195^

In light of all these developments, a critical question is how likely is it that someone will try to reidentify the source of a deidentified sample. Recent investigations have suggested that in many circumstances, it simply may not be worth the attacker's while to identify someone from his or her deidentified DNA, given the costs of attempting to do so, especially if the biobank protects the data.^196^ Even less is known about the circumstances under which an attacker would seek to reidentify DNA in order to learn about the individual's genetic traits and predispositions, especially since that information might be more easily available in other ways. Nonetheless, efforts may be warranted to create incentives to decrease the probability of reidentification as well as to ameliorate any adverse consequences that might occur were inappropriate identification to occur. Part of the solution to deter reidentification in the first place may be to adopting the proposal by the Working Group of the Precision Medicine Initiative^197^ that Congress adopt penalties for inappropriately reidentifying or otherwise misusing data.

### VI.B. Surreptitious Genetic Testing

Surreptitious or nonconsensual genetic testing refers to the covert collection and analysis of an individual's genetic material without their consent, generally carried out by another individual, such as a family member or a current/former romantic partner, or by law enforcement in the forensic context. Individuals may have a variety of motives for surreptitious genetic testing, such as to covertly determine parentage, to uncover whether a romantic partner is being unfaithful, or to discover sensitive medical information such as disease or carrier status, perhaps about a potential partner. In the law enforcement context, police use surreptitious forensic testing as an investigatory tool to gather evidence against an individual suspected of a crime and to facilitate identification of a suspect.^198^ Whether carried out by a private citizen or law enforcement, each of these developments raises their own set of unique ethical issues and privacy concerns.

The rise in surreptitious testing has been made possible by the increasing sensitivity and availability (and decreasing cost) of genetic testing and analysis. Numerous studies^199^ have documented the proliferation of companies offering these services directly to consumers and, in some cases, law enforcement. A recent survey of 90 DTC companies operating in the USA revealed that nearly one-third appeared to offer some form of surreptitious testing, generally alongside paternity and other family relationship tests. Companies offer these services under a variety of different names (eg ‘forensic’, ‘discreet’, ‘special sample’, and ‘infidelity’ testing) and permit, or even encourage, consumers to submit covertly collected samples ranging from strands of hair, discarded cigarette butts, and used condoms to articles of clothing containing suspicious stains.^200^ However, companies rarely warn consumers of the potential legal consequences that might arise from the surreptitious collection or analysis of another person's genetic material without their consent and often have privacy policies lacking even the basic information about their practices regarding the collection, use, and sharing of genetic data.^201^

The most obvious issue raised by surreptitious testing, generally in the context of testing performed by private citizens, is the lack of consent. Knowledgeable agreement to be tested is vital due to the potentially harmful consequences that could flow from the unwanted disclosure of that information (eg disruption of family relationships stemming from misattributed parentage, unwanted revelations regarding cultural/racial identity, or discrimination based on disease or carrier status) as well as the potential secondary uses of the genetic information once it enters the DTC ecosystem (where it could be used for internal research and product development by the company, or shared with third parties for research, commercial, or law enforcement purposes). Given the likely motives for surreptitious testing and its connection to the paternity and family relationship testing industry, the practice is likely to implicate the genetic material of children/minors. A less obvious concern, present in both the civilian and law enforcement contexts, relates to the underlying quality of the samples being analysed. Unlike testing performed on samples collected in more controlled settings, surreptitious testing generally involves analysis of samples containing DNA of questionable quality or in limited quantity, greatly increasing the possibility of erroneous results, which might have serious consequences for the individual being tested.

Data on how frequently individuals engage in surreptitious testing are sparse, but a recent survey of Canadian consumers of DTC services provides some insight into the frequency with which individuals submit the genetic material of others for testing.^202^ The study found that one-third of consumers who had purchased DTCs (60 of 180) reported that they had submitted the sample of another person for testing, with or without consent, including their children or their partner's children, current and past partners, suspected children or parents, or other family members.^203^ Over a third of these individuals (38%; 23 of 60) reported that they had not obtained permission before submitting the person's sample for testing and analysis.^204^ While the study's authors noted that the apparent lack of permission does not necessarily imply nefarious intent or that the test was carried out in a truly surreptitious fashion (eg parents obtaining testing on behalf of their child or a willing family member), these figures raise serious questions and concerns about the prevalence of this practice.

The frequency with which surreptitious testing appears to occur might not be surprising in light of the paucity of relevant federal and state law on the subject and the limited scope of the laws that do exist. Despite repeated calls from legal scholars^205^ and government advisory committees^206^ for increased oversight of surreptitious testing and stricter laws governing nonconsensual collection and analysis of the genetic material of others, no comprehensive federal laws currently prohibit the practice. However, federal laws, such as the ACA^207^ or GINA,^208^ may provide some limited protection against the practice if it were to be undertaken to limit access to employment or health insurance. In contrast, the UK recognizes ‘DNA theft’ as a crime, punishable by a monetary fine and/or up to three years of imprisonment, which strictly prohibits individuals from analysing the genetic material of others without their consent in many circumstances.^209^

Instead, the USA relies on a patchwork of state laws that place varying restrictions on the practice depending on the purpose of the testing or the context in which it is performed. A 50-state survey conducted by the Genetics and Public Policy Center in 2009 revealed that a total of 29 states had laws ‘restrict[ing] collection of DNA samples, DNA analysis, [and/or] disclosure of test results without the consent of the person tested.’^210^ Surreptitious genetic testing performed for health-related purposes was the most commonly restricted activity (15 states), although a number of states placed restrictions on nonconsensual testing for both health-related and non-health-related purposes (10 states).^211^ A subset of states restricted surreptitious testing only when carried out in a specific context, such as court-ordered parentage proceedings (six states) or employment (two states).^212^ The possible penalties varied widely by state, ranging from exposure to civil liability in a private cause of action to criminal punishment in the form of fines (generally ranging from $1000 to $10,000) and/or sentences of up to one year in jail. Still unclear is the extent to which courts will be willing to recognize a property interest in genetic material sufficient to support causes of action for surreptitious testing under common-law torts such as conversion or invasion of privacy.^213^

The result of this heterogeneity is that DTC companies are left to set their own policies governing surreptitious testing and the submission of another individual's sample without their consent. According to the Best Practices recently developed (and adopted) by industry leaders in conjunction with the Future of Privacy Forum, companies should require separate express consent from consumers that are submitting samples on behalf of others.^214^ Specifically, companies are encouraged to adopt policies that ‘require that the individual submitting the Biological Sample or the Genetic Data is the owner or include reasonable steps to ensure that consent has been obtained from the owner of the Biological Sample or Genetic Data.’^215^ It remains to be seen whether DTC companies, particularly those that permit or even encourage consumers to surreptitiously submit samples as a key component of their business model, will adopt this practice, and if they do, what steps they will take to ensure that the individual submitting the sample has obtained consent to do so.

Surreptitious testing by law enforcement agencies also raises privacy concerns, an issue that has gained renewed attention in the wake of revelations surrounding the arrest of the suspected Golden State Killer. ^216^ After homing in on the suspect using familial searching of an open-access genealogy website, investigators were able to verify his identity by analysing DNA surreptitiously collected from a car door handle, taken while the suspect shopped, and later from a discarded tissue found in the trash outside of his home. While it appears that investigators in this case obtained a court order before performing this surreptitious testing (although not before searching the genealogy website), police in many jurisdictions are not required to seek approval from a court before engaging in this practice.^217^

Police have this freedom because the state laws that place restrictions on surreptitious testing generally do not apply to surreptitious forensic testing,^218^ and the Fourth Amendment has thus far provided little protection in the context of surreptitious genetic testing by law enforcement. Although the Supreme Court has not specifically ruled on the issue of surreptitious genetic testing, it has established that individuals have no reasonable expectation of privacy in abandoned property.^219^ While several states have held that placing items for trash pickup does not amount to a complete abandonment of any interest in the contents,^220^ police can engage in surreptitious DNA collection and analysis without a warrant or a court order in most circumstances.^221^ In the absence of constitutional or statutory prohibitions, the prevalence of surreptitious testing by law enforcement will only continue to increase.

## VII. CONCLUSION

In this article, we have focused primarily on issues of genetic privacy in the context of healthcare, but our analysis necessarily addresses health information more generally as well. A lot of health information provides insights or at least clues into the individual's genetic makeup, so that the two cannot readily be separated. Moreover, a person's current condition or phenotype can be more pertinent to privacy concerns than his or her genes. Thus, treating genetic data as exceptional, as deserving special protection, is generally unwarranted and in many cases not achievable or even counterproductive.^222^

Concerns about genetic privacy and health information privacy more broadly fall into two large categories—the ability to control where data about individuals go and the extent to which individuals can be assured that data about them will not be used to cause them harm.^223^ Our analysis, which focuses on the role of law, goes primarily to the question of how much control people have, and concludes that control is limited in many ways. In the healthcare system, patients are asked to sign an acknowledgement of a covered entity's notice of privacy practices when they seek care, which may lead them to believe that their health privacy is vigorously protected, but the law's protection may be illusory. The HIPAA Privacy Rule has numerous exceptions permitting access to individually identifiable health information, which reflect policy trade-offs between individual control and social uses. But until recently, even when these exceptions were invoked, there was little risk that genetic information would be shared because personally identified health information rarely contained much genetic data.

One incontestable fact is that the landscape is evolving as more genetic and genomic data are becoming available. Within the healthcare system, more genetic tests are coming into clinical use, increasingly using broad-based platforms with the capacity to uncover variants potentially pertinent to conditions beyond the initial clinical indication. Although healthcare institutions have and will continue to have strong incentives to protect patients’ information due to the increasing emphasis on transparency and trust, once in the patient's medical record a wide range of entities may be granted access to genetic information pursuant to broad regulatory exceptions under the HIPAA Privacy Rule.^224^ In addition, millions of people are compelled every year to provide unlimited access to their health information for various uses, such as insurance and commercial transactions.^225^ To the extent that these data become available outside healthcare institutions (ie HIPAA covered entities), it loses even the little protection afforded by the HIPAA Privacy Rule, creating the possibility for harm or misuse by an array of downstream actors.

A crucial change in the ecology of genetic information is the emergence of DTC genetic testing and interpretation, so far used by millions of people and largely escaping regulation, except in some cases when these companies offer to provide health-related results. The most common use by far is to explore one's ancestral origins and to find relatives. The latter use necessarily requires identifiable genetic information in order to make or disprove relationships. The most prominent of these companies have explicit privacy policies and usually require people to give permission before they are placed in contact with a putative relative.^226^ Others say little or nothing at all about privacy. Many companies encourage surreptitious testing. Clearly, there is room here to require more robust privacy policies that allow people to decide whether they want to communicate with a purported relative and to forbid surreptitious testing.

One of the most significant challenges is that many people take genetic data about themselves, which they often received from DTC companies, and post them online in an identifiable form to find their relatives, to share with other people with similar conditions, or to promote research. These actions necessarily reveal information about their relatives, as has been made clear by the use of GEDMatch to identity criminal suspects. At present, a person has no ability to prevent his or her relatives from revealing their own information. Moreover, there are no limits on who can access these data or for what purpose.

Our research has demonstrated that increasing amounts of genetic information are generated, analysed, shared, and stored by diverse individuals and entities. The HIPAA Privacy Rule was never intended to afford comprehensive health privacy protection. Even when health information is stored at compliant healthcare institutions, the combination of broad exceptions and compelled disclosures precludes informational health privacy.

At the same time that genetic information is flowing through covered entities’ sieve-like regulatory structures, many other entities that obtain sensitive health information are unregulated. The latter group varies widely in the extent to which they are likely to protect data about a person, which depends on their motives and business models.

Other disclosures of genetic information occur when individuals voluntarily make their identified genomic data public; in many cases, people do this without considering or regardless of the impact on themselves or their relatives. There is little that can be done to prevent these voluntary disclosures except to ensure that individuals are aware of the possible consequences.

Our overview of the law of genetic privacy has been quite sobering. Although some opportunities exist to increase individual control over disclosures that may affect them, these situations are limited. Thus, it may be time to shift attention from attempting to control access to genetic information to considering the more challenging question of how these data can be used and under what conditions, explicitly addressing trade-offs between individual and social goods in numerous applications. The first step to meaningful protection of genetic privacy may be the societal recognition that health privacy, including genetic privacy, is now largely a mirage.

## Footnotes

^1^ Even the genomes of monozygotic (‘identical’) twins often differ in some ways. See, eg F. Nipa Haque, Irving I. Gottesman & Albert H.C. Wong, *Not Really Identical: Epigenetic Differences in Monozygotic Twins and Implications for Twin Studies in Psychiatry*, 151C Am. J. Med. Genetics Part C Semin. Med. Genetics 136 (2009).

^2^ Human Subjects Research Protections: Enhancing Protections for Research Subjects and Reducing Burden, Delay, and Ambiguity for Investigators, 76 Fed. Reg. 143 (proposed July 26, 2011) (to be codified at 45 C.F.R. pts. 46, 160, 164; 21 C.F.R. pts. 50, 56).

^3^
Francis S. Collins, The Language of Life: DNA and the Revolution in Personalized Medicine (2010).

^4^ Ruth R. Faden et al., *An Ethics Framework for a Learning Healthcare System: A Departure from Traditional Research Ethics and Clinical Ethics*, 43 Hastings Ctr. Rep. S16, S23 (2013).

^5^ Samuel D. Warren & Louis D. Brandeis, *The Right to Privacy*, 4 Harv. L. Rev. 193, 193 (1890).

^6^ Vera Bergelson, *It's Personal but Is It Mine? Toward Property Rights in Personal Information*, 37 U.C. Davis L. Rev. 379, 401–2 (2003); Daniel J. Solove, *Conceptualizing Privacy*, 90 Calif. L. Rev. 1087, 1092–1126 (2002).

^7^ Bergelson, *supra* note 6, at 401 [quoting Raymond T. Nimmer, The Law of Computer Technology ¶ 16.02, at 16-5 (2001)].

^8^ Paul M. Schwartz, *Internet Privacy and the State*, 32 Conn. L. Rev. 815, 820 (2000).

^9^ See, eg U.S. Dep’t of Health and Human Servs., *Standards for Privacy of Individually Identifiable Health Information*, 65 Fed. Reg. 82,462, 82,606 (Dec. 28, 2000) (noting, in the preamble to the original HIPAA Privacy Rule, that various industry and standard-setting organizations have recognized the need for individual access, stating that, ‘Patients’ confidence in the protection of their information requires that they have the means to know what is contained in their records’).

^10^ Laurie D. Smith, Laurel K. Willig & Stephen F. Kingsmore, *Whole-Exome Sequencing and Whole-Genome Sequencing in Critically Ill Neonates Suspected to Have Single-Gene Disorders*, 6 Cold Spring Harbor Persp. Med. 2 (2016).

^11^ Jonathan S. Berg, Muin J. Khoury & James P. Evans, *Deploying Whole Genome Sequencing in Clinical Practice and Public Health: Meeting the Challenge One Bin at a Time*, 13 Genetics Med. 499 (2011)

^12^ Nanibaa’A. Garrison et al., *A Systematic Literature Review of Individuals' Perspectives on Broad Consent and Data Sharing in the United States*, 18 GeneticsMed. 663, 668–9 (2016); C. Sanderson et al., *Public Attitudes Toward Consent and Data Sharing in Biobank Research: A Large Multi-site Experimental Survey in the US*, 100 Am. J. Hum. Genetics 414, 421 (2017).

^13^ Ellen W. Clayton et al., *A Systematic Literature Review of Individuals’ Perspectives on Privacy and Genetic Information in the United States*, Plos One,https://doi.org/10.1371/journal.pone.0204417 (2018); Stacey Pereira et al., *Do Privacy and Security Regulations Need a Status Update? Perspectives from an Intergenerational Study*, Plos One, https://doi.org/10.1371/journal.pone.0184525 (2017).

^14^ Mary Madden, *Public Perceptions of Privacy and Security in the Post-Snowden Era*, Pew Res. Ctr.,http://www.pewinternet.org/2014/11/12/public-privacy-perceptions/(2014).

^15^
Philip Reilly, Genetics, Law, and Social Policy (1977); Genetic Secrets: Protecting Privacy and Confidentiality in the Genomic Era (Mark A. Rothstein ed., 1997).

^16^ For recent discussions, see Josephine Johnston, Ruth M.Farrell & Eric Parens, *Supporting Women's Autonomy in Prenatal Testing,* 377 New Eng. J. Med. 505 (2017); Ruth M. Farrell & Megan A. Allyse, *Key Ethical Issues in Prenatal Genetics,*45 Obstet. & Gynecol. Clin. 127 (2017).

^17^ Many other countries, especially those in the European Union, use the term ‘data protection’ as an omnibus concept that includes privacy, confidentiality, security, and other elements. These concepts are at the heart of the European Union's General Data Protection Regulation, which took effect in 2018. General Data Protection Regulation, 2018 O.J. (L 127), https://gdpr-info.eu (accessed Apr. 15, 2019). See generally Edward S. Dove, *The EU General Data Protection Regulation: Implications for International Scientific Research in the Digital Era,* 46 J.L. Med. & Ethics, 1013-30 (2018).

^18^ ‘Physical and informational privacy practices serve to limit observation and disclosure deemed inimical to well-being’. Anita L. Allen, *Privacy in Health Care*, *in* 4 Encyclopedia of Bioethics 2067 (Warren Thomas Reich ed., 1995).

^19^ Anita L. Allen, *Genetic Privacy: Emerging Concepts and Values*, *in*Genetic Secrets: Protecting Privacy and Confidentiality in the Genetic Era 31, 33 (Mark A. Rothstein ed., 1997).

^20^ See *infra* Section V.

^21^ See Bartha Maria Knoppers & Madelaine Saginur, *The Babel of Genetic Data Terminology*, 23 Nature Biotech. 925, 925 (2005) (discussing the numerous terms used to describe measures to protect genetic information).

^22^ ‘Confidentiality concerns the communication of private and personal information from one person to another where it is expected that the recipient of the information, such as a health professional, will not ordinarily disclose the confidential information to third persons’. William J. Winslade, *Confidentiality*, *in* 1 Encyclopedia of Bioethics at 452 (Warren Thomas Reich ed., 1995). See also Mark A. Rothstein, *Confidentiality*, *in*Medical Ethics: Analysis of the Issues Raised by the Codes, Opinions, and Statements 171 (Baruch A. Brody et al. eds., 2001).

^23^ For a further discussion, see *infra* Section III.

^24^ For example, laws requiring the reporting of infectious diseases or suspected cases of child abuse to appropriate governmental agencies override confidentiality.

^25^ See 45 C.F.R. pt. 164 (2018) (security and privacy provision of the HIPAA Privacy Rule). See generally Sharona Hoffman & Andy Podgurski, *In Sickness, Health and Cyberspace: Protecting the Security of Electronic Private Health Information*, 48 B.C. L. Rev. 331 (2007); Nicolas P. Terry & Leslie P. Francis, *Ensuring the Privacy and Confidentiality of Electronic Health Records*, 2007 U. Ill. L. Rev 681 (2007).

^26^ See Ellen Wright Clayton & Bradley Malin, *Assessing Risks to Privacy in Biospecimen Research*, in Specimen Science: Ethics and Policy Implications 143 (Holly Fernandez Lynch et al. eds., 2017); Sara Renee Savage, *Characterizing the Risks and Harms of Linking Genetic Information to Individuals*, 15 IEEE Security & Privacy 14, 16 (2017). For a further discussion, see Part VI-A.

^27^ Jennifer Kulynych & Henry T. Greely, *Clinical Genomics, Big Data, and Electronic Medical Records: Reconciling Patient Rights with Research When Privacy and Science Collide*, J.L. & Biosciences 94 (2017); Mark A. Rothstein, *Is Deidentification Sufficient to Protect Health Privacy in Research?*, 10 Am. J. Bioethics 3 (2010).

^28^ See generally Sarah E. Igo, The Known Citizen: A History of Privacy in Modern America (2018).

^29^ See, eg the Fair Credit Reporting Act of 1970, 15 U.S.C. § 1681b (enumerating permissible disclosure of people's credit information and conditions for such disclosures); Privacy Act of 1974, 5 U.S.C. § 552a(b) (requiring governmental agencies to seek consent prior to disclosure of people's personal data stored in governmental databases, but then allowing various enumerated exceptions to the consent requirement); HIPAA Privacy Rule, 45 C.F.R. § 164.512 (allowing unconsented use and disclosure of people's health and genetic information for an enumerated list of purposes—such as public health, law enforcement and judicial uses, and research subject to IRB or privacy board approval).

^30^
Privacy Protection Study Commission, Personal Privacy in an Information Society (July, 1977), https://www.ncjrs.gov/pdffiles1/Digitization/49602NCJRS.pdf (accessed Apr. 15, 2019).

^31^
*Id*. at 299*.* See Margaret O’Mara*, The End of Privacy Began in the 1960s,*N.Y. Times, Dec. 6, 2018, at A31 (stating that as early as the 1960s Congress adopted the policy of pushing for data transparency, including sharing data with the person the data describe, rather than restrictions on sharing people's data with third parties).

^32^ See, eg the Privacy Act of 1974, 5 U.S.C. 552a(d)(1) (granting an individual right of access to certain data held in governmental databases); HIPAA Privacy Rule, 45 C.F.R. § 164.524 (granting an individual right of access to certain data held by HIPAA-covered entities). See also European Union General Data Protection Regulation (Regulation (EU) 2016/679), Art. 15 (providing an individual access right).

^33^ See Carolyn T. Lye et al., *Assessment of US Hospital Compliance with Regulations for Patients’ Requests for Medical Records*, 1 JAMA Network Open. e183014 (2018), DOI:10.1001/jamanetworkopen.2018.3014 (finding widespread noncompliance with federal regulations by 83 hospitals studied).

^34^ Under the HIPAA Privacy Rule, individuals may request that their health records be revised or supplemented, but covered entities are not required to do so. 45 C.F.R. § 164.526. As a practical matter, covered entities often fail to grant such requests by patients.

^35^ Thomas H. Murray, *Genetic Exceptionalism and ‘Future Diaries’: Is Genetic Information Different from Other Medical Information?*, in Genetic Secrets: Protecting Privacy and Confidentiality in the Genetic Era (Mark A. Rothstein ed., 1997). See also Nicolas P. Terry, *Big Data Proxies and Health Privacy Exceptionalism*, 24 Health Matrix 65 (2014) (discussing the broader ‘health privacy exceptionalism’).

^36^ Murray, *supra* note 35, at 71. Although Mendelian conditions, especially Huntington disease, were cited extensively in the literature in the 1990s as justifying genetic exceptionalism, it is not a good example upon which to construct an approach to genetic ethics and policy. For example, few other genetic conditions share the characteristics of Huntington disease, which is an autosomal dominant, progressive, neurological disorder with nearly complete penetrance, adult onset, and usually resulting in death within 12 to 15 years of onset. Jean Paul G. Vansattel & Marian DiFiglia, *Huntington Disease*, 57 J. Neuropathol. & Exp. Neurol. 369, 369 (1998).

^37^ For example, family health history information often contains genetic information and is widely dispersed in health records. Similarly, patients’ own histories may imply much about their genetic makeup.

^38^ ‘Genetic reductionism, understood ontologically, is the position that organisms consist of nothing but genes’. Robert Wachboit, *Genetic Determinism, Genetic Reductionism, and Genetic Essentialism*, in 1 Encyclopedia of Ethical, Legal, and Policy Issues in Biotechnology 353, 354 (Thomas H. Murray & Maxwell J. Mehlman, eds., 2000). See also Richard M. Lerner, *Eliminating Genetic Reductionism from Developmental Science*, 12 Res. Human Dev. 178 (2015).

^39^ ‘The phrase “genetic determinism” would, strictly speaking, mean that every event has a genetic cause that is sufficient for that event's occurring’. Wachbroit, *supra* note 38, at 353. See also Emily Willoughby et al., *Free Will, Determinism, and Intuitive Judgments about the Heritability of Behavior,*Behav. Genetics (2018), https://doi.org/10.1007/s10519-018-9931-1.

^40^ See Lawrence O. Gostin & James G. Hodge, Jr, *Genetic Privacy and the Law: An End to Genetics Exceptionalism*, 40 Jurimetrics J. 21, 23 (1999); Deborah Hellman, *What Makes Genetic Discrimination Exceptional?*, 29 Am. J.L. & Med. 77, 83 (2003); Trudo Lemmens, *Selective Justice, Genetic Discrimination, and Insurance: Should We Single Out Genes in Our Laws?*, 45 McGill L.J. 347, 369–76 (2002); Mark A. Rothstein & Mary R. Anderlik, *What Is Genetic Privacy, and When and How Should It Be Prevented?*, 3 Genetics Med. 354 (2001); Sonia M. Suter, *The Allure and Peril of Genetics Exceptionalism: Do We Need Special Genetics Legislation?*, 79 Wash. U. L.Q. 669 (2001). For publications proposing separate treatment of genetics, see George J. Annas et al., The Genetic Privacy Act and Commentary pt. D, § 131(e)(1)(B) (1995); Colin S. Diver & Jane Maslow Cohen, *Genophobia: What Is Wrong with Genetic Discrimination?*, 149 U. Pa. L. Rev. 1439, 1454–59 (2001); Robert M. Green & A. Mathew Thomas, *DNA: Five Distinguishing Features for Policy Analysis*, 11 Harv. J.L. & Tech. 571, 572 (1998).

^41^ In the 1970s, Florida, Fla. Stat. § 448.075 (2018); Louisiana, La. Stat. Ann. §§ 23:1001 to :1004 (2018); and North Carolina, N.C. Gen. Stat. § 95-28.1 (2018) enacted laws prohibiting genetic discrimination in health insurance. In 1981, New Jersey enacted a broader law prohibiting discrimination based on an individual's ‘atypical hereditary cellular or blood type’, defined to include sickle cell trait, hemoglobin C trait, thalassemia trait, Tay Sachs trait, or cystic fibrosis trait. N.J. Stat. Ann. § 10:5-5(y) (1981).

^42^ Leslie E. Wolf et al., *The Web of Legal Protections for Participants in Genomic Research* (forthcoming 2019).

^43^ Pub. L. 110–233, 122 Stat. 881 (May 21, 2008), 42 U.S.C. § 2000ff (2018).

^44^ 42 U.S.C. §§ 18001–18122 (2018). The Health Insurance Portability and Accountability Act, initially enacted in 1996, prohibited exclusion from employer-sponsored group health plans on the basis of genetic conditions, but its protection was limited by its failure to prohibit differential rates. Other laws, such as the Americans with Disabilities Act, also provide some protection to those who are severely affected by genetic disorders. Ellen W. Clayton, *Why the Americans with Disabilities Act Matters for Genetics*, 313 JAMA 2225, 2225–6 (2014).

^45^ Mark A. Rothstein, *Genetic Exceptionalism and Legislative Pragmatism*, 35 Hastings Ctr. Rep. 27, 31 (2005).

^46^ See *infra* Section IV.

^47^ See *infra* Section V. See *infra* Section VI for a discussion of the issue of the use of data from identifiers that have been removed.

^48^ As discussed below, under the HIPAA Privacy Rule, disclosures of protected health information for treatment need not be limited in scope and do not require consent or authorization.

^49^ Improper disclosures and uses of genetic information also may take place in research settings, such as where (1) genetic information is used for research without consent or beyond the bounds of the consent; (2) genetic information specifically stored in a deidentified form is reidentified without authorization or a legitimate purpose; (3) genetic information is used for research that is objectionable to the individual; and (4) genetic information is used for research with the potential to cause group harms.

^50^ See Andrea Gurmankin Levy et al., *Prevalence of and Factors Associated with Patient Nondisclosure of Medically Relevant Information to Clinicians*, 1 JAMA Netw. Open. e185293 (2018), DOI:10.1001/jamanetworkopen.2018.5293 (reporting on a survey showing that various privacy concerns caused many patients to avoid telling clinicians information about their health).

^51^ 42 U.S.C. §§ 300gg-300gg-2 (2018).

^52^ 45 C.F.R. pts. 160, 162, 164 (2018).

^53^ See the HIPAA statute, §§ 261–264 (enacting a new part C of title IX of the Social Security Act).

^54^ HIPAA statute, § 264(c).

^55^
*Id*.

^56^
*Id.* § 160.103.

^57^ The 2013 and 2014 amendments to the Privacy Rule incorporated provisions mandated by the Health Information Technology for Economic and Clinical Health Act (HITECH Act), American Recovery and Reinvestment Act of 2009, Pub. L. No. 111-5, tit. XII, 123 Stat. 115, 203–226, and the Genetic Information Nondiscrimination Act (GINA). Another shortcoming of the Privacy Rule is that it does not provide for private actions to redress harms caused by violations. The Privacy Rule merely provides that a person who believes a covered entity is not complying with applicable requirements of the Privacy Rule may file a complaint with the Secretary of Health and Human Services. 45 C.F.R. § 160.306 (2018).

^58^ 45 C.F.R. § 160.103 (2018).

^59^ See GINA § 102 [amending the Public Health Service Act at 42 U.S.C. § 300gg-91(d)(16) to define ‘genetic information’ very broadly as including ‘with respect to any individual, information about – (i) such individual's genetic tests, (ii) the genetic tests of family members of such individual, and (iii) the manifestation of a disease or disorder in family members of such individual’ and further including ‘genetic services and participation in genetic research’]. See also *id*. at § 300gg-91(d)(17) (defining ‘genetic test’ as meaning ‘an analysis of human DNA, RNA, chromosomes, proteins, or metabolites, that detects genotypes, mutations, or chromosomal changes’ and thus clearly including non-clinically-significant information, such as raw genomic data, within the scope of information included in GINA’s definition of ‘genomic information’) and see *id.* at § 300gg-91(d)(18) [defining ‘genetic services’ as including genetic tests and ‘genetic counseling (including obtaining, interpreting, or assessing genetic information)’ and genetic information, such that information from testing, assessing, and counseling occurring during the course of genetic research is included in GINA’s broad definition of ‘genetic information’] and see GINA § 105 (adding a new § 1180 to the Social Security Act, 42 U.S.C.A. § 1320d-9, providing that ‘[t]he Secretary shall revise the HIPAA privacy regulation’ so that ‘[g]enetic information shall be treated as health information described in section 1320d(4)(B) of this title’, which was the section of the Social Security Act added by the 1996 HIPAA statute in which Congress defined the ‘health information’ that is subject to HIPAA’s privacy protections). And see GINA § 105.

^60^
*Id.* § 164.514(g).

^61^
*Id.* § 164.502(a)(5)(i); § 164.520(b)(1)(iii)(C).

^62^ Only psychotherapy notes receive special treatment in the Privacy Rule. Separately maintained notes of private communication are not considered part of the designated record set that may be disclosed for treatment, payment, or healthcare operations. *Id.* § 164.501*.*

^63^ See GINA § 105, *supra* note 59.

^64^
*Id.* § 164.103. See the Modifications to the HIPAA Privacy, Security, Enforcement, and Breach Notification Rules, 78 Fed. Reg. 5566, 5661 (2013) (codified at 45 C.F.R. pts. 160, 164).

^65^ Protected health information generally includes individually identifiable health information. 45 C.F.R. § 160.103 (2018).

^66^
*Id.* § 164.502(a)(1)(ii). The Privacy Rule defines treatment, payment, and healthcare operations quite broadly, and therefore covered entities may use and disclose numerous types of PHI without consent or additional notice to the individual beyond the notice of privacy practices.

^67^
*Id.* § 164.520.

^68^ 45 C.F.R. § 164.528 (2018).

^69^
*Id.* § 164.514(d)(3)(i).

^70^ See Julie L. Agris, *Extending the Minimum Necessary Standard to Uses and Disclosures for Treatment*, 42 J.L. Med. & Ethics 263, 264 (2014). Despite its manifest inadequacies, the Privacy Rule has some value, including the following: (1) it provides individuals with a right of access to their health records, *id.* § 164.524, an especially valuable provision in states lacking similar state legislation; (2) it requires authorizations for uses and disclosures of PHI in fundraising, *id.* § 164.514(f), marketing, *id.* § 164.508(a)(3), and most research, *id.* § 164.512(i); and (3) it has substantial symbolic value by declaring the importance of health information privacy and security, eg, banning healthcare providers from discussing patients’ health information in public areas.

^71^ See Mark A. Rothstein, *The End of the HIPAA Privacy Rule?*, 44 J.L. Med. & Ethics 352, 353 (2016) (advocating for adoption of a ‘least identifiable form’ requirement under the Privacy Rule).

^72^
*Genome Statute and Regulation Database*, Nat’l Hum. Genome Res. Inst. (NHGRI),https://www.genome.gov/policyethics/legdatabase/pubsearchresult.cfm (accessed Nov. 2, 2018).

^73^ 42 U.S.C. § 2000ff.

^74^ See Jessica L. Roberts, *Preempting Discrimination: Lessons from the Genetic Information Nondiscrimination Act*, 63 Vand. L. Rev. 439 (2010).

^75^ See Mark A. Rothstein, *GINA at Ten and the Future of Genetic Nondiscrimination Law*, 48 Hastings Ctr. Rep. No. 3, at 5 (2018).

^76^ GINA, Pub. L. No. 110-233, § 101(d), 122 Stat. 881, 884–5 (2008) (prohibiting acquisition of genetic information by ERISA-qualified health plans); § 102(d)(2)(A), 122 Stat. at 896 (prohibiting acquisition of genetic information by group health plans or group health insurers); § 102(d)(2)(B), 122 Stat. at 896 (prohibiting acquisition of genetic information in individual health insurance); § 103(d), 122 Stat. at 898–9 (amending the Internal Revenue Code to prohibit acquisition of genetic information with regard to group premiums); § 104(b)(2), 122 Stat. at 901 (prohibiting acquisition of genetic information in regard to Medigap policies).

^77^ GINA § 102, *supra* note 59.

^78^ The original HIPAA Privacy Rule, which became effective in 2003–04, only protected ‘health information’ as defined by Section 1171 of the Social Security Act, 42 U.S.C.A. § 1320d(4). This implied that genetic information was protected by the Privacy Rule if it was ‘**(A)** created or received by a health care provider, health plan, public health authority, employer, life insurer, school or university, or health care clearinghouse; and **(B)** relates to the past, present, or future physical or mental health or condition of an individual, the provision of health care to an individual, or the past, present, or future payment for the provision of health care to an individual’. Non-medical genetic information (such as forensic identifiers or variant data having no established clinical significance) seemingly was not protected by the Privacy Rule. When Congress enacted GINA, Congress defined ‘genetic information’ broadly, as discussed earlier in note 59. See the Public Health Service Act § 2791(d)(16), codified at 42 U.S.C. 300gg–91(d)(16) (defining genetic information as including information about a person's genetic tests, tests of family members, and manifest disease in family members, and including genetic services and participation in genetic research). GINA added a new Section 1180 to the Social Security Act, 42 U.S.C.A. § 1320d-9, which deems all such ‘genetic information’ to meet the definition of ‘health information’, for purposes of the HIPAA Privacy Rule. After GINA, even non-clinically significant genetic information, such as forensic data, is treated as ‘health information’ for purposes of being protected under the Privacy Rule, even if it would not be considered ‘health information’ in other legal contexts.

^79^ 45 C.F.R. § 160.306 (2018). See also Acara v. Banks, 470 F.3d 569, 571-72 (5th Cir. 2006) (holding, in the first federal appellate decision to address this issue, that the Privacy Rule does not create a private right of action).

^80^ Robert C. Green et al., *ACMG Recommendations for Reporting of Incidental Findings in Clinical Exome and Genome Sequencing*, 15 Genetics Med. 565, 569–573 (2013).

^81^ See Wylie Burke et al., *Recommendations for Returning Genomic Incidental Findings? We Need to Talk!*, 15 Genetics Med. 854, 855 (2013); Lainie F. Ross et al., *Mandatory Extended Searches in All Genome Sequencing: “Incidental Findings,” Patient Autonomy, and Shared Decision Making*, 310 Jama 367, 368 (2013).

^82^ S. S. Kalia et al., *Recommendations for Reporting of Secondary Findings in Clinical Exome and Genome Sequencing, 2016 Update (ACMG SFv.2.0): A Policy Statement of the American College of Medical Genetics and Genomics*, 19 Genetics Med. 249, 250 (2017).

^83^ Safer v. Estate of Pack, 677 A.2d 1188 (N.J. Super. Ct. App. Div.), *cert. denied*, 683 A.2d 1163 (N.J. 1996). The holding in this case has never been cited with approval and was severely limited by the New Jersey legislature. See N.J. Rev. Stat. § 10:5-47 (2018).

^84^ American Society of Human Genetics Social Issues Subcommittee on Familial Disclosure, *Professional Disclosure of Familial Genetic Information*, 62 Am. J. Hum. Genetics 474, 474 (1998). The exceptional circumstances justifying an otherwise impermissible disclosure are described as follows: Disclosure should be permissible where attempts to encourage disclosure on the part of the patient have failed; where the harm is highly likely to occur and is serious and foreseeable; where the at-risk relative (s) is identifiable; and where either the disease is preventable/treatable or medically accepted standards indicate that early monitoring will reduce the genetic risk. *Id.* at 474.

^85^ The HIPAA Privacy Rule contains an exception that permits the following disclosure: ‘Uses and disclosures to avert a serious threat to health or safety’. 45 C.F.R. § 164.512(j) (2018). This provision was intended to apply to situations, such as the *Tarasoff* case, where an individual disclosed to his psychotherapist that he intended to kill a female acquaintance. Tarasoff v. Regents of the Univ. of Cal., 551 P.2d 334, 339 (Cal. 1976). See Office for Civil Rights, Department of Health and Human Services, FAQ: Does HIPAA permit a doctor to contact a patient's family or law enforcement if the doctor believes that the patient might hurt herself or someone else? https://www.hhs.gov/hipaa/for-professionals/faq/2096/does-hipaa-permit-doctor-contact-patients-family-or-law-enforcement-if-doctor-believes-patient.html. (‘The Privacy Rule permits a healthcare provider to disclose necessary information about a patient to law enforcement, family members of the patient, or other persons, when the provider believes the patient presents a serious and imminent threat to self or others’.). See also Mark A. Rothstein, *Tarasoff Duties after Newtown,* 42 J.L. Med. & Ethics 104 (2014). Therefore, the ‘serious threat to health or safety’ exception does not apply to warnings by a healthcare provider to a patient's relatives regarding their genetic risk. In 2013, the Office for Civil Rights of the Department of Health and Human Services issued the following interpretation: ‘Health care providers may share genetic information about an individual with providers treating family members of the individual who are seeking to identify their own genetic risks, provided that the individual has not agreed to a restriction on such disclosure’. Modifications to the HIPAA Privacy, Security, Enforcement and Breach Notification Rules, 78 Fed. Reg. 5566, 5668 (2013). Although this interpretation permits the release of sensitive information without the consent of the patient, the interpretation is limited. Healthcare providers are not required to make such disclosures, and they may make them only to another healthcare provider, and only in response to an inquiry by another healthcare provider.

^86^ See Mark A. Rothstein, *Reconsidering the Duty to Warn Genetically At-Risk Relatives*, 19 Genetics Med. 285, 288–9 (2018).

^87^ See R.R. Fabsitz et al., *Ethical and Practical Guidelines for Reporting Research Results to Study Participants: Updated Guidelines from a National Heart, Lung, and Blood Institute Working Group*, 3 Circ. & Cardiovasc. Genet. 574, 574–580 (2010); Susan M. Wolf et al., *Returning a Research Participant's Genomic Results to Relatives: Analysis and Recommendations*, 43 J.L. Med. & Ethics 440, 445–6, 451 (2015).

^88^ See James W. Hazel & Christopher Slobogin, *Who Knows What, and When?: A Survey of the Privacy Policies Proffered by U.S. Direct-to-Consumer Genetic Testing Companies*, 28 Cornell J.L. & Pub. Pol’y 35, 47 (2018); Andelka M. Phillips, *Only a Click Away—DTC Genetics for Ancestry, Health, Love…and More: A View of the Business and Regulatory Landscape*, 8 Appl. & Transl. Genom. 16, 16–9 (2016).

^89^
*Id*.

^90^
Rosanna Hertz & Margaret K. Nelson, Random Families: Genetic Strangers, Sperm Donor Siblings, and the Creation of New Kin (2019).

^91^
*Woman Uses DNA Test, Finds Sperm Donor – and Pays a ‘Devastating’ Price* (CBS News 31 Jan. 2019), https://www.cbsnews.com/news/woman-finds-sperm-donor-after-using-dna-test-raising-questions-about-donor-anonymity/ (accessed Mar. 11, 2019) (woman sued by the sperm bank for breach of contract by accidentally identifying the donor).

^92^ Press Release, *FDA Permits Marketing of First Direct-to-Consumer Genetic Carrier Test for Bloom Syndrome* (FDA, Feb. 19, 2015), https://wayback.archive-it.org/7993/20170111191740/http://www.fda.gov/NewsEvents/Newsroom/PressAnnouncements/ucm435003.htm (accessed Apr. 15, 2019).

^93^ Press Release, *FDA Allows Marketing of First Direct-to-Consumer Tests that Provide Genetic Risk Information for Certain Conditions* (FDA, Apr. 6, 2017), https://www.fda.gov/newsevents/newsroom/pressannouncements/ucm551185.htm (accessed Apr. 15, 2019).

^94^ Press Release, *FDA Authorizes, with Special Controls, Direct-to-Consumer Test that Reports Three Mutations in the BRCA Breast Cancer Genes* (FDA, Mar. 6, 2018), https://www.fda.gov/NewsEvents/Newsroom/PressAnnouncements/ucm599560.htm (accessed Apr. 15, 2019).

^95^ Press Release, *Statement from FDA Commissioner Scott Gottlieb, M.D., on Implementation of Agency's Streamlined Development and Review Pathway for Consumer Tests that Evaluate Genetic Health Risks* (FDA, Nov. 6, 2017), https://www.fda.gov/NewsEvents/Newsroom/PressAnnouncements/ucm583885.htm (accessed Apr. 15, 2019).

^96^ Press Release, *FDA Authorizes First Direct-to-Consumer Test for Detecting Genetic Variants that May Be Associated with Medication Metabolism* (FDA, Oct. 31, 2018), https://www.fda.gov/NewsEvents/Newsroom/PressAnnouncements/ucm624753.htm (accessed Apr. 15, 2019).

^97^ Hazel & Slobogin, *supra* note 88, at 48–57.

^98^ See *id*. (discussing ‘catch-all’ provisions that appear to permit sharing with third parties other than law enforcement in many circumstances, including to protect the rights of the company, other users, or the public, or to enforce the company's terms and conditions).

^99^ Health Insurance Portability and Accountability Act of 1996, Pub. L. No. 104-191, 110 Stat. 1936.

^100^ Clinical Laboratory Improvement Amendments of 1988, Pub. L. No. 100-578, 102 Stat. 2903 (codified at 42 U.S.C. § 263a (2018)).

^101^ Helen C. Dick, *Risk and Responsibility: State Regulation and Enforcement of the Direct-to-Consumer Genetic Testing Industry*, 6 St. Louis U.J. Health L. & Pol’y 167, 174–184 (2012).

^102^ See Jessica L. Roberts, *Progressive Genetic Ownership*, 93 Notre Dame L. Rev. 1105, 1128 (2018) (discussing five states that recognize such a property interest: Alaska, Colorado, Florida, Georgia, and Louisiana).

^103^ The most notable example is California's Online Privacy Protection Act of 2003 (CalOPPA), a law that requires commercial websites that collect personal information from California consumers to post a privacy policy detailing what information is being collected and with what third parties it is being shared. See Cal. Bus. & Prof. Code §§ 22575-22579 (2004).

^104^ Cole v. Gene by Gene, Ltd., No. 1:14-cv-00004, 2017 U.S. Dist. LEXIS 101761 at ^*^7, 9 (D. Alaska June 30, 2017) (denying the defendant's motion to dismiss on the ground that Cole was able to demonstrate the requisite injury-in-fact under Alaska's Genetic Privacy Act, which ‘recognizes an exclusive property interest in one's DNA, and prohibits the unauthorized disclosure of DNA information’); Cole v. Gene by Gene, Ltd., No. 17-35837 at ^*^2 (9th Cir. Aug. 21, 2018) (affirming the trial court's denial of class certification for 900 Gene by Gene, Ltd. customers because ‘Cole failed to show that ‘common questions . . . predominate over any questions affecting only individual members’ of his proposed class and subclass’). See also Roberts, *supra* note 102, at 1110 (describing the factual background underlying the *Cole* case).

^105^ Andelka M. Phillips, *Reading the Fine Print When Buying Your Genetic Self Online: Direct-to-Consumer Genetic Testing Terms and Conditions*, 36 New Genetics & Soc’y 273, 282 (2017).

^106^ Federal Trade Commission Act of 1914, Ch. 311, § 5, 38 Stat. 719 (codified as amended at 15 U.S.C. §§ 41–58 (2018)).

^107^ Complaint at 10–11, *In re* GeneLink, Inc. & Foru Int’l Corp., No. 112-3095 (F.T.C. Jan. 7, 2014), https://www.ftc.gov/system/files/documents/cases/140512genelinkcmpt.pdf (accessed Apr. 15, 2019).

^108^
*Privacy Online: Fair Information Practices in the Electronic Marketplace*, U.S. Fed. Trade Commission (May 2000), https://www.ftc.gov/sites/default/files/documents/reports/privacy-online-fair-information-practices-electronic-marketplace-federal-trade-commission-report/privacy2000.pdf (describing ‘the four widely-accepted fair information practices’ of Notice, Choice, Access and Security). See also Robert Gellman, Fair Information Practices: A Basic History (Apr. 10, 2017) (unpublished manuscript), https://bobgellman.com/rg-docs/rg-FIPshistory.pdf (accessed Apr. 15, 2019) (describing the various articulations of the fair information practices by various government agencies, including the FTC).

^109^
*Protecting Consumer Privacy in an Era of Rapid Change*, U.S. Fed. Trade Commission (May 2012), https://www.ftc.gov/sites/default/files/documents/reports/federal-trade-commission-report-protecting-consumer-privacy-era-rapid-change-recommendations/120326privacyreport.pdf (accessed Apr. 15, 2019) (urging companies operating online to adopt the principles of ‘Privacy by Design’, ‘Simplified Consumer Choice’, and ‘Transparency’).

^110^ Press Release, *Future of Privacy Forum and Leading Genetic Testing Companies Announce Best Practices to Protect Privacy of Consumer Genetic Data* (Future of Privacy Forum, July 31, 2018), https://fpf.org/2018/07/31/future-of-privacy-forum-and-leading-genetic-testing-companies-announce-best-practices-to-protect-privacy-of-consumer-genetic-data/ (accessed Apr. 15, 2019).

^111^
*Privacy Best Practices for Consumer Genetic Testing Services*, Future of Privacy Forum (July 31, 2018), https://fpf.org/wp-content/uploads/2018/07/Privacy-Best-Practices-for-Consumer-Genetic-Testing-Services-FINAL.pdf (accessed Apr. 15, 2019). Key recommendations found in the Best Practices document include (1) ‘Detailed transparency about how Genetic Data is collected, used, shared, and retained including a high-level summary of key privacy protections posted publicly and made easily accessible to consumers’; (2) ‘Separate express consent for transfer of Genetic Data to third parties and for incompatible secondary uses’; (3) ‘Educational resources about the basics, risks, benefits, and limitations of genetic and personal genomic testing’; (4) ‘Access, correction, and deletion rights’; (5) ‘Valid legal process for the disclosure of Genetic Data to law enforcement and transparency reporting on at least an annual basis’; (6) ‘Ban on sharing Genetic Data with third parties (such as employers, insurance companies, educational institutions, and government agencies) without consent or as required by law’; (7) ‘Restrictions on marketing based on Genetic Data’; and (8) ‘Strong data security protections and privacy by design, among others’. Press Release, *supra* note 110.

^112^
Future of Privacy Forum, *supra* note 111, at 3 (noting, however, ‘that currently, Genetic Data held *at the individual-level* that has been deidentified cannot be represented as strongly protecting individuals from reidentification, based upon existing deidentification tools and standards. Such data may be protected in other ways and used for research with appropriate consent and security controls’).

^113^
*Id.* at 9.

^114^ See, eg Complaint at 12–13*, In re* GeneLink, *supra* note 107.

^115^ Mark A. Rothstein & Meghan K. Talbott, *Compelled Disclosures of Health Records: Updated Estimates*, 45 J.L. Med. & Ethics 149 (2017).

^116^ 45 C.F.R. § 164.512(a) (2018).

^117^
*Id.* § 164.512(b).

^118^
*Id.* § 164.512(c).

^119^
*Id.* § 164.512 (d).

^120^
*Id.* § 164.512 (e).

^121^
*Id.* § 164.512 (f).

^122^
*Id.* § 164.512 (g).

^123^
*Id.* § 164.512 (h).

^124^
*Id.* § 164.512 (i).

^125^
*Id.* § 164.512 (j).

^126^
*Id.* § 164.512 (k).

^127^
*Id.* § 164.512 (l).

^128^ See Rothstein & Talbott, *supra* note 115.

^129^ See Rothstein & Anderlik, *supra* note 40, at 152.

^130^ 42 U.S.C. § 14132 (2018). See *Frequently Asked Questions on CODIS and NDIS*, FBI, http://www.fbi.gov/services/laboratory/biometric-analysis.codis/codis-and-ndis-fact-sheet (accessed Mar. 5, 2018).

^131^ Mark A. Rothstein & Meghan K. Talbott, *The Expanding Use of DNA in Law Enforcement: What Role for Privacy?*, 34 J.L. Med. & Ethics 153, 154 (2006).

^132^ See, eg J.W. Hazel et al., *Is it Time for a Universal Genetic Forensic Database?*, 362 Science 898 (2018); David H. Kaye & Michael E. Smith, *DNA Identification Databases: Legality, Legitimacy, and the Case for Population-Wide Coverage*, 2003 Wis. L. Rev. 413, 415 (2003); Arnold H. Loewy,*A Proposal for the Universal Collection of DNA*, 48 Tex. Tech. L. Rev. 261, 262 (2015).

^133^ See, eg Henry T. Greely et al., *Family Ties: The Use of DNA Offender Databases to Catch Offenders’ Kin*, 34 J.L. Med. & Ethics 248, 253–4 (2006); David H. Kaye, *Trawling DNA Databases for Partial Matches: What Is the FBI Afraid of?*, 19 Cornell J.L. & Pub. Pol’y 145, 166 (2009).

^134^ See Mark A. Rothstein, *Applications of Behavioural Genetics: Outpacing the Science?*, 6 Nature Revs. Genetics 793, 794–5 (2005).

^135^ 45 C.F.R. § 164.512(f) (2018).

^136^ 20 U.S.C. § 1232(g) (2018).

^137^
*Id*. §§ 1400–1482.

^138^
*Id.* §§ 12101-12213.

^139^
*Id.* § 706(8)(B).

^140^ See Laura F. Rothstein, *Genetic Information in Schools*, in Genetic Secrets: Protecting Privacy and Confidentiality in the Genetic Era 317–331 (Mark A. Rothstein ed., 1997).

^141^ 42 U.S.C. § 2000ff (2018).

^142^ See Robert C. Green et al., *GINA, Genetic Discrimination, and Genomic Medicine*, 372 New Eng. J. Med. 397, 397 (2015); Jessica L. Roberts, *Rethinking Employment Discrimination Harms*, 91 Ind. L.J. 393, 401 (2016); Jessica L. Roberts, *Preempting Discrimination: Lessons from the Genetic Information Nondiscrimination Act*, 63 Vand. L. Rev. 439, 443 (2010); Mark A. Rothstein, Jessica L. Roberts, & Tee L. Guidotti, *Limiting Occupational Medical Examinations under the Americans with Disabilities Act and the Genetic Information Nondiscrimination Act*, 41 Am. J.L. & Med. 523, 550–1 (2015).

^143^ 42 U.S.C. § 12112(d)(4) (2018); 29 C.F.R. § 1630.14(b)(3) (2018).

^144^ See Rothstein, Roberts & Guidotti, *supra* note 142.

^145^
*See* Mark A. Rothstein, *GINA, the ADA, and Genetic Discrimination in Employment*, 36 J.L. Med. & Ethics 837, 839 (2008). With regard to [their] coverage, GINA and the ADA are mirror images, with GINA covering asymptomatic individuals and the ADA covering those with conditions that have manifested. The problem is that there is a large gap between these statutes, and some individuals may not be covered under either law. This would include individuals who have a biomarker of genome-environment interaction, a subclinical marker of aberrant gene expression, or an initial symptom of a gene-associated disease. New medical technologies, laboratory tests, and sophisticated imaging that measure incipient or occult disease based on gene-mediated processes could increase this gap. Mark A. Rothstein, *GINA at Ten and the Future of Genetic Nondiscrimination Law*, 48 Hastings Ctr. Rep. 5, 6 (2018).

^146^ NHGRI, *supra* note 72.

^147^
*See* Genetic Ties and the Family: The Impact of Paternity Testing on Parents and Children (Mark A. Rothstein et al. eds., 2005).

^148^
*See, eg* Atcherian v. State, 14 P.3d 970, 973 (Alaska 2000) (holding that man was entitled to a refund only of child support paid after date he filed motion to vacate default judgment of paternity on basis of genetic test results); *In re* Parentage of C.S., 139 P.3d 366, 368 (Wash. Ct. App. 2006) (holding that husband was not permitted to disestablish himself as presumed parent and to establish paternity of another man).

^149^
*Uniform Parentage Act*, Uniform Law Commission,http://www.uniformlaws.org/Committee.aspx?title=Parentage%20Act%20(2017) (accessed Mar. 12, 2018).

^150^ In EEOC v. Burlington N. Santa Fe R.R. Co., No. C01-4013 (N.D. Iowa, filed Feb. 9, 2001), the EEOC sought a preliminary injunction against the railroad to end the required genetic testing of employees who filed claims for work-related injuries based on carpal tunnel syndrome. The EEOC alleged that the employees were unaware that required blood samples were used for a genetic test for a chromosome 17 deletion associated with hereditary pressure palsy neuropathy, a rare condition that may predict some forms of carpal tunnel syndrome. The action was based on section 102(d)(4) of the Americans with Disabilities Act, 42 U.S.C. § 12112(d)(4), which provides that any medical examinations or inquiries of current employees must be either voluntary or job-related. The EEOC’s position was that the unproven genetic test was not job-related. The case was subsequently settled, with the company discontinuing its practice of genetic testing.

^151^
*Declaration of Human Rights*, United Nations Gen. Assembly,http://www.un.org/en/documents/udhr (accessed Mar. 13, 2018).

^152^ See Palmira Granados Moreno & Yann Joly, *DNA Testing for Immigration and Family Reunification?*, J.L. & Biosci.,http://blog.oup.com/2017/08/dna-testing-immigration-family-reunification/ (accessed Apr. 15, 2019) (2017) (noting that at least 21 countries, including Austria, Canada, Finland, France, Germany, UK, and USA, use DNA testing in family reunification cases). See also J. Taitz et al., *The Last Resort: Exploring the Use of DNA Testing for Family Reunification*, 6 Health & Hum. Rts. 20, 24 (2002); Torsten Heinemann & Thomas Lemke, *Biological Citizenship Reconsidered: The Use of DNA Analysis by Immigration Authorities in Germany*, 39 Sci. Tech. & Hum. Values 488, 496 (2014).

^153^
Lori Andrews & Dorothy Nelkin, Body Bazaar: The Market for Human Tissue in the Biotechnology Age 115-16 (2001).

^154^
NIH-DOE Working Group on Ethical, Legal, and Social Implications of Human Genome Research, Genetic Information and Health Insurance (1993).

^155^ All states except Mississippi and Pennsylvania have enacted such a law. See NHGRI, *supra* note 72.

^156^ 42 U.S.C. §§ 18001-18122 (2018).

^157^ NHGRI, *supra* note 72.

^158^ See Genetics and Life Insurance: Medical Underwriting and Public Policy (Mark A. Rothstein ed., 2004); Anya E. R. Prince, *Insurance Risk Classification in an Era of Genomics: Is a Rational Discrimination Policy Rational?*, 96 Neb. L. Rev. 624, 644–5 (2018); Mark A. Rothstein, *Time to End the Use of Genetic Test Results in Life Insurance Underwriting*, 46 J.L. Med. & Ethics 794, 797 (2018).

^159^ See Susan M. Wolf & Jeffrey P. Kahn, *Genetic Testing and the Future of Disability Insurance: Ethics, Law & Policy*, 35 J.L. Med. & Ethics 6, 7 (2007) (Supp. 2).

^160^ See Mark A. Rothstein, *Predictive Genetic Testing for Alzheimer's Disease in Long-Term Care Insurance*, 35 Ga. L. Rev. 707, 725 (2001); Donald H. Taylor, Jr. et al., *Genetic Testing for Alzheimer's and Long-Term Care Insurance*, 29 Health Aff. 102, 105 (2010); Cathleen Zick et al., *Genetic Testing for Alzheimer's Disease and Its Impact on Insurance Purchasing Behavior*, 24 Health Aff. 483, 484 (2005).

^161^ For example, Vermont prohibits insurance companies from requiring genetic testing as a condition of applying for any type of insurance as well as using the results of genetic tests of family members in underwriting. Insurers may still use the results of an applicant's genetic tests performed in the clinical setting and documented in his or her medical record. Vt. Stat. Ann. tit. 18, § 9334(a).

^162^ See National Research Council, Applications of Toxicogenomic Technologies to Predictive Toxicology and Risk Assessment (2007).

^163^ See Genomics and Environmental Regulation: Science, Ethics, and Law (Richard R. Sharp et al. eds., 2008); Andrew Gendron & Thomas Morgan, *Whole Exome Sequencing and Federal Courts*, For the Defense, Jan. 2019, at 22.

^164^ Section 202(b)(5) of GINA permits an employer to conduct genetic monitoring of the biological effects of toxic substances in the workplace, but only if the employer (A) provides written notice of the monitoring; (B) the employee provides prior, knowing, voluntary, and written authorization or the monitoring is required by federal or state law; (C) the employee is informed of individual monitoring results; (D) the monitoring complies with any federal or state regulations dealing with genetic monitoring; and (E) the employer, excluding any licensed healthcare professional or certified genetic counselor, receives the results only in aggregate form and does not disclose the identity of specific employees. This exception applies only to the monitoring of current employees and does not permit the testing of applicants or conditional offerees for genetic susceptibility to workplace exposures. For a pre-GINA proposal recommending voluntary genetic testing and monitoring before and after the commencement of employment, see Mark A. Rothstein, *Genetics and the Work Force of the Next Hundred Years*, 3 Colum. Bus. L. Rev. 371, 393–5 (2000).

^165^ See Jamie A. Grodsky, *Genomics and Toxic Torts: Dismantling the Risk-Injury Divide*, 59 Stan. L. Rev. 1671, 1693 (2007); Gary E. Marchant, *Genetic Data in Toxic Tort Litigation*, 14 J.L. & Pol’y 7, 9–10 (2006). See also Gary E. Marchant et al., From Genetics to Genomics: Facing the Liability Implications (2019).

^166^ See Diane E. Hoffmann & Karen H. Rothenberg, *Judging Genes: Implications of the Second Generation of Genetic Tests in the Courtroom*, 66 Md. L. Rev. 858, 865 (2007); Anthony S. Niedwiecki, *Science Fact or Science Fiction? The Implications of Court-Ordered Genetic Testing Under Rule 35*, 34 U. S. Fla. L. Rev. 295, 296 (2000); Mark A. Rothstein, *Preventing the Discovery of Plaintiff Genetic Profiles by Defendants Seeking to Limit Damages in Personal Injury Litigation*, 71 Ind. L.J. 877, 881–2 (1996).

^167^ See Mark A. Rothstein & Laura Rothstein, *How Genetics Might Affect Real Property Rights*, 44 J.L. Med. & Ethics 216, 217 (2016).

^168^ Fair Housing Amendments Act of 1988, Pub. L. No. 100-430, 102 Stat. 619 (codified as amended at 42 U.S.C. §§ 3601–3631 (2018)).

^169^
Cal. Gov’t Code §§ 12920–12922, 12955 (West 2018).

^170^ 21st Century Cures Act, Pub. L. No. 114-255, 102 Stat. 1033 (2016).

^171^
*Notice of Changes to NIH Policy for Issuing Certificates of Confidentiality*, Nat’l Insts. Health (2017), https://grants.nih.gov/grants/guide/notice-files/NOT-OD-17-109.html (accessed Apr. 15, 2019).

^172^ Jeffrey R. Botkin et al., *Privacy and Confidentiality in the Publication of Pedigrees: A Survey of Investigators and Biomedical Journals*, 279 JAMA 1808, 1812 (1998).

^173^ 45 C.F.R. § 46.101(b)(4) (2018).

^174^ Oscar Ferrandez et al., *Evaluating Current Automatic De-identification Methods with Veteran's Health Administration Clinical Documents*, 12 BMC Med. Res. Methodology 109 (2012), at 1, 2; Sharona Hoffman & Andy Podgurski, *Balancing Privacy, Autonomy, and Scientific Needs in Electronic Health Records Research*, 65 SMU L. Rev. 85, 95 (2012).

^175^ Zhen Lin et al., *Genetics, Genomic Research and Human Subject Privacy*, 305 Science 183, 183 (2004); Bradley Malin & Latanya Sweeney, *How (Not) to Protect Genomic Data Privacy in a Distributed Network: Using Trail Re-identification to Evaluate and Design Anonymity Protection Systems*, 37 J. Biomedical Informatics 179, 191 (2004).

^176^ Gymrek et al., *Identifying Personal Genomes by Surname Inference*, 339 Science 321, 321 (2013).

^177^ Jill Pulley et al., *Principles of Human Subjects Protections Applied in an Opt-out De-identified Biobank,* 3 Clin. Transl. Sci. 42 (2010).

^178^ Victor W. Weedn & Howard J. Baum, *DNA Identification in Mass Fatality Incidents*, 32 Am. J. ForensicMed. Pathology 393, 393 (2011); Frederick R. Bieber et al., *Human Genetics. Finding Criminals through DNA of Their Relatives*, 312 Science 1315, 1315 (2006).

^179^ There may be more overlap between forensic and research data in the future. Jaehee Kim et al., *Statistical Detection of Relatives Typed with Disjoint Forensic and Biomedical Loci*, 175 Cell 848 (2018).

^180^
*Transparency Report*, 23andMe,https://www.23andme.com/transparency-report/; https://www.ancestry.com/cs/transparency (accessed Oct. 15, 2018).

^181^ Hazel & Slobogin, *supra* note 88, at 30–1.

^182^ Madeline P. Ball et al., *Harvard Personal Genome Project: Lessons from Participatory Public Research*, Genome Med., 2014, at 1, 1; Madeline P. Ball et al., *A Public Resource Facilitating Clinical Use of Genomes*, 109 Proc. Nat’l Acad. Scis. U.S. 11920, 11920 (2012).

^183^ B. Greshake et al., *OpenSNP–A Crowdsourced Web Resource for Personal Genomics*, PLOS One, 2014, at 1, 2, https://doi.org/10.1371/journal.pone.0089204 (accessed Apr. 15, 2019).

^184^ Tobias Haeusermann et al., *Open Sharing of Genomic Data: Who Does It and Why?*, PLOS One, 2017, at 1, 2, https://doi.org/10.1371/journal.pone.0177158 (accessed Apr. 15, 2019).

^185^
*Tools for DNA and Genealogy Research*, GED Match,www.gedmatch.com (accessed Nov. 2, 2018).

^186^ Doc Edge & Graham Coop, *How Lucky Was the Genetic Investigation in the Golden State Killer Case?*, WordPress: Gcbias (May 7, 2018),https://gcbias.org/2018/05/07/how-lucky-was-the-genetic-investigation-in-the-golden-state-killer-case/ (accessed Apr. 15, 2019).

^187^
*Updates to the Terms of Service and Privacy Policy at GEDmatch*, Blogger: Cruwys News (May 21, 2018), https://cruwys.blogspot.com/2018/05/updates-to-terms-of-service-and-privacy.html (accessed Apr. 15, 2019).

^188^ Natalie Ram et al., *Genealogy Databases and the Future of Criminal Investigation*, 360 Science 1078, 1078 (2018).

^189^ Kristen V. Brown, *Killer App? DNA Site Had Unwitting Role in Golden State Manhunt*, Bloomberg (May 29, 2018, 4:00 PM), https://www.bloomberg.com/news/articles/2018-05-29/killer-app-dna-site-had-unwitting-role-in-golden-state-manhunt (accessed Apr. 15, 2019).

^190^
Blogger: Cruwys News, *supra* note 187.

^191^ Christoph Lippert et al., *Identification of Individuals by Trait Prediction Using Whole-Genome Sequencing Data*, 114 Proc. Nat’lAcad. Scis. U.S. 10166, 10169 (2017).

^192^ Antonio Regalado, *Does Your Genome Predict Your Face? Not Quite Yet*, MIT Tech. Rev. (Sept. 7, 2017), https://www.technologyreview.com/s/608813/does-your-genome-predict-your-face-not-quite-yet/ (accessed Apr. 15, 2019).

^193^ Gretchen Vogel, *In Germany, Controversial Law Gives Bavarian Police New Power to Use DNA*, Science (May 15, 2018, 5:20 PM), http://www.sciencemag.org/news/2018/05/germany-controversial-law-gives-bavarian-police-new-power-use-dna (accessed Apr. 15, 2019).

^194^ Latanya Sweeney et al., *Identifying Participants in the Personal Genome Project by Name (A Re-identification Experiment)*, ArXiV (Apr. 29, 2013), https://arxiv.org/abs/1304.7605 (accessed Apr. 15, 2019).

^195^ This may be particularly concerning in the case where one party receives genomic data on a limited number of individuals about whom the first party has other information that could be used to facilitate reidentification. Leslie E. Wolf et al., *Certificates of Confidentiality: Protecting Human Subject Research Data in Law and Practice*, 43 J.L. Med. & Ethics 594, 594 (2015).

^196^ Zhiyu Wan et al., *Expanding Access to Large-Scale Genomic Data While Promoting Privacy: A Game Theoretic Approach*, 100 Am. J. Hum. Genetics 316, 317 (2017).

^197^ NIH Precision Medicine Initiative (PMI) Working Group Report to the Advisory Committee to the Director, *The Precision Medicine Initiative Cohort – Building a Research Foundation for 21st Century Medicine*, nih (2015), http://acd.od.nih.gov/reports/DRAFT-PMI-WG-Report-9-11-2015-508.pdf (accessed Apr. 15, 2019).

^198^ Rothstein & Talbott, *supra* note 131, at 156.

^199^ See Hazel & Slobogin, *supra* note 88, at 48, 56–7; Emily Christofides & Kieran O’Doherty, *Company Disclosure and Consumer Perceptions of the Privacy Implications of Direct-to-Consumer Genetic Testing*, 35 New Genetics & Soc’y 101, 107 (2016); Andelka M. Phillips, *Only a Click Away—DTC Genetics for Ancestry, Health, Love…and More: A View of the Business and Regulatory Landscape*, 8 Appl. & Transl. Genom. 16, 17, 22 (2016).

^200^ Hazel & Slobogin, *supra* note 88, at 14.

^201^
*Id.* at 14–5*.*

^202^ Christofides & O’Doherty, *supra* note 199, at 112–3.

^203^
*Id.* at 113. Interestingly, no participant surveyed in this study reported using these services to discover infidelity.

^204^
*Id.*

^205^ See, eg Elizabeth Joh, *DNA Theft: Recognizing the Crime of Nonconsensual Genetic Collection and Testing*, 91 B.U.L. REV. 665, 668–9 (2011); Mark A. Rothstein, *Genetic Stalking and Voyeurism: A New Challenge to Privacy*, 57 Kan. L. Rev. 539, 561–2 (2009); Nicole Strand, *Shedding Privacy Along with Our Genetic Material: What Constitutes Adequate Legal Protection Against Surreptitious Genetic Testing?*, 18 AMA J. Ethics 264, 274 (2016).

^206^ See, eg Sec’y's Advisory Comm. on Genetics, Health, & Soc’y, U.S. Dep’t of Health & Human Servs., Direct-to-Consumer Genetic Testing(Apr. 2010), https://osp.od.nih.gov/wp-content/uploads/2013/11/SACGHS_DTC_Report_2010.pdf (accessed Apr. 15, 2019) (identifying ‘the extent to which DTC services are being used for surreptitious genetic testing’ as an area warranting additional scrutiny by the committee and federal agencies).

^207^ 42 U.S.C. §§ 18001-18122 (2018).

^208^ Genetic Information Non-Discrimination Act, Pub. L. No. 110–233, 122 Stat. 881 (2008).

^209^ U.K. Human Tissue Act of 2004.

^210^
*State Laws Pertaining to Surreptitious DNA Testing*, Genetics & Pub. Pol’y Ctr. (2009),https://web.archive.org/web/20100827054416/http://www.dnapolicy.org/resources/State_law_summaries_final_all_states.pdf.

^211^
*Id.* at 1*.*

^212^
*Id.*

^213^ See, eg Peerenboom v. Perlmutter, No. 2013-CA-015257 (Fla. Cir. Ct. Jan. 23, 2017); Roberts, *supra* note 102, at 1109–1110 (discussing *Pereenboom*, a highly publicized, ongoing case originating in Florida involving a claim of conversion for surreptitious testing). In *Peerenboom*, Isaac and Laura Perlmutter sued Harold Peerenboom for conversion, among other things. They alleged that Peerenboom conspired to obtain their genetic material as part of a scheme to retaliate against the Perlmutters in a neighborhood dispute. The Perlmutters asserted that they ‘have an exclusive right of possession and ownership of the genetic information encoded in their genetic material’ and that ‘[b]y collecting, analyzing, and testing their genetic material to obtain the Perlmutters’ confidential genetic information, Conspirators exercised an act of dominion and authority that deprived the Perlmutters of their rights of ownership, possession, control, and privacy’. Responding to a motion to dismiss from Peerenboom, the trial court found that the Perlmutters enjoyed a property right in their genetic information, sufficient to state a claim for conversion. Roberts, *supra* note 102, at 1109–1110 (citations omitted).

^214^
Future of Privacy Forum, *supra* note 111, at 4–5.

^215^
*Id.* at 4–5.

^216^ Nancy Dillon, *Golden State Killer Suspect Arrested After Cops Swiped His DNA from Car Door Handle and Tissue*, N.Y. Daily News (June 1, 2018), http://www.nydailynews.com/news/crime/ny-news-golden-state-killer-dna-collected-car-door-trash-20180601-story.html (accessed Apr. 15, 2019).

^217^ Albert E. Scherr, *Genetic Privacy and the Fourth Amendment: Unregulated Surreptitious DNA Harvesting*, 47 Ga. L. Rev. 445, 525 (2013).

^218^
*Id.*

^219^ California v. Greenwood, 486 U.S. 35, 40–41 (1988) (holding there is no reasonable expectation of privacy for garbage left at curbside).

^220^ State v. Tanaka, 701 P.2d 1274, 1276-77 (Haw. 1985) (stating that ‘[p]eople reasonably believe that police will not indiscriminately rummage through their trash bags to discover their personal effects’); State v. Goss, 834 A.2d 316, 317-19 (N.H. 2003) (holding there was a reasonable expectation of privacy in black plastic trash bags in driveways on trash days); State v. Hempele, 576 A.2d 793, 800-02 (N.J. 1990) (suppressing evidence from search of white plastic trash bags in plastic garbage in front of house); State v. Morris, 680 A.2d 90, 94 (Vt. 1996) (holding warrant needed to search secure opaque bags left for pickup). *Contra* People v. Hillman, 834 P.2d 1271, 1276 (Colo. 1992) (holding society does not recognize ‘as reasonable an expectation of privacy in garbage left adjacent to a public sidewalk for collection’); Commonwealth v. Pratt, 555 N.E.2d 559, 567-68 (Mass. 1990) (holding no reasonable expectation of privacy in trash left for collection); State v. Carriere, 545 N.W.2d 773, 776 (N.D. 1996) (same).

^221^ See Scherr, *supra* note 217. See also Joh, *supra* note 205, at 696.

^222^ See Part II-B.

^223^ Clayton et al., *supra* note 13, at 17.

^224^ See Part V-A.

^225^ See Part V-B*.*

^226^ Hazel & Slobogin, *supra* note 88, at 21.

